# Mesenteric adipose-derived exosomal TINAGL1 enhances intestinal fibrosis in Crohn's Disease via SMAD4

**DOI:** 10.1016/j.jare.2024.05.016

**Published:** 2024-05-13

**Authors:** Yidong Chen, Junrong Li, Xiaopeng Zhang, Shuang Li, Yiyu Cheng, Xiaoyu Fu, Jiamin Li, Liangru Zhu

**Affiliations:** Division of Gastroenterology, Union Hospital, Tongji Medical College, Huazhong University of Science and Technology, Wuhan 430022, China

**Keywords:** Crohn's Disease, Intestinal Fibrosis, Exosomes, TINAGL1, TGF-β/SMAD4 Signaling

## Abstract

**Introduction:**

Crohn's Disease (CD) is a chronic inflammatory condition characterized by intestinal fibrosis, severely impacting patient quality of life. The molecular mechanisms driving this fibrosis remain inadequately understood. Recent evidence implicates mesenteric adipose tissue (MAT) in CD pathogenesis, particularly through its exosome secretion, which may influence fibrogenic pathways. Understanding the role of MAT-derived exosomes is crucial for unraveling these molecular processes.

**Objectives:**

This study aims to elucidate the role of MAT-derived exosomes in CD-related intestinal fibrosis. We focus on investigating their molecular composition and the potential impact on fibrosis progression, with an emphasis on identifying novel therapeutic targets.

**Methods:**

We induced chronic intestinal inflammation in mice using dinitrobenzene sulfonic acid (DNBS), simulating CD-like fibrosis. Exosomes were isolated from DNBS-treated mice (MG) and normal controls (NG) for characterization using electron microscopy and proteomic analysis. Additionally, human colonic fibroblasts were exposed to exosomes from CD patients and healthy individuals, with subsequent assessment of fibrogenesis through proteomic and RNA sequencing analyses.

**Results:**

Proteomic analyses revealed a significant activation of the TGF-β signaling pathway in MG-treated mice compared to controls, correlating with enhanced intestinal fibrosis. In vitro experiments demonstrated that colonic fibroblasts exposed to CD patient-derived exosomes exhibited increased fibrogenic activity. Protein docking and co-immunoprecipitation studies suggested a critical interaction between TINAGL1 and SMAD4, enhancing fibrosis. Importantly, in vivo experiments corroborated that recombinant TINAGL1 protein exacerbated DNBS-induced intestinal fibrosis.

**Conclusion:**

Our findings highlight the pivotal role of MAT-derived exosomes, particularly those carrying TINAGL1, in the progression of intestinal fibrosis in CD. The involvement of the TGF-β signaling pathway, especially the SMAD4 protein, offers new insights into the molecular mechanisms of CD-related fibrosis and presents potential targets for therapeutic intervention.

## Highlights

• **Novel Insights into Fibrogenesis:** Our study reveals the critical role of TINAGL1, enriched in mesenteric adipose tissue (MAT)-derived exosomes, in promoting intestinal fibrosis in Crohn's Disease.
• **TINAGL1-SMAD4 Interaction:** We demonstrate a significant interaction between TINAGL1 and the transcription factor SMAD4, highlighting a previously unexplored pathway in fibrogenesis, with implications for the exacerbation of Crohn's Disease.
• **Potential for Therapeutic Targeting:** The findings offer a new avenue for therapeutic strategies, suggesting that targeting the TINAGL1-SMAD4 axis could mitigate the fibrotic complications in Crohn's Disease.
• **Broadening Understanding of Exosomes:** Our work adds to the growing body of evidence that underscores the importance of exosomes as key mediators in cellular communication and disease pathogenesis, extending beyond gastrointestinal disorders.

## Graphical abstract

Elevated TINAGL1 levels in exosomes from mesenteric adipose tissue (MAT) significantly influence Crohn's Disease progression. The visual abstract depicts how TINAGL1, upon interacting with SMAD4 within these exosomes, activates colonic fibroblasts, thereby exacerbating intestinal fibrosis. This illustration connects the rise in TINAGL1 to subsequent cellular activations and pathological changes in the intestines, emphasizing the crucial role of exosomal TINAGL1 in advancing the disease.

**Figure:**
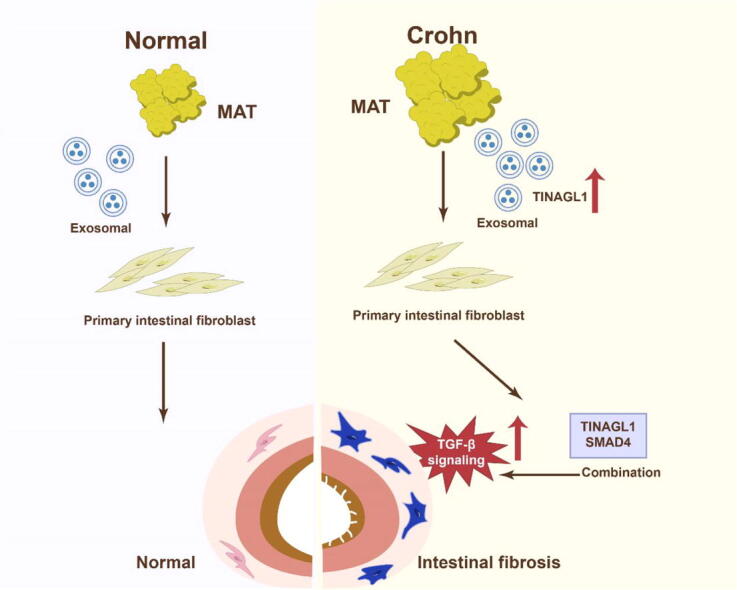

## Introduction

Crohn's Disease (CD), a form of Inflammatory Bowel Disease (IBD), presents a significant global health challenge, affecting millions worldwide [1]. Characterized by chronic inflammation of the gastrointestinal tract, CD manifests with diverse clinical symptoms including abdominal pain, diarrhea, and weight loss [2]. The disease's intermittent nature, marked by periods of flare-ups and remission, profoundly impacts the quality of life of patients [3]. The etiology of CD is multifactorial, encompassing genetic, environmental, and immunological factors, contributing to its complex clinical management [4].

A debilitating complication of CD is intestinal fibrosis, a process characterized by the excessive deposition of extracellular matrix components, leading to intestinal strictures and obstruction [5]. Alarmingly, about one-third of CD patients develop intestinal fibrosis, with approximately three-quarters of these cases necessitating surgical intervention [6]. This underscores the significant clinical burden of fibrosis in CD. Central to this fibrotic process are fibroblasts, which, when activated in response to inflammation or injury, proliferate and produce large amounts of extracellular matrix proteins [7].

Despite advancements in understanding the inflammatory aspects of CD, the specific mechanisms driving fibroblast activation and the subsequent fibrosis remain insufficiently elucidated. The current therapeutic options for managing fibrotic complications in CD are limited, largely due to a lack of clarity regarding the molecular pathways involved in fibroblast modulation [8]. This gap in knowledge highlights the urgent need for research focused on unraveling the underlying mechanisms of intestinal fibrosis in CD, including the role of fibroblasts. Such understanding could pave the way for novel therapeutic strategies.

Mesenteric adipose tissue (MAT) in Crohn's Disease represents a dynamic entity, comprising not just adipocytes but also inflammatory cells, reflecting its active involvement in the intestinal environment [9], [10]. MAT's response to CD-associated inflammation can lead to its transformation into 'creeping fat', where it extensively surrounds inflamed intestinal segments, particularly near fibrotic areas [11], [12]. This transition of MAT underscores its potential role in the process of fibrogenesis. Despite its observed protective role in containing inflammation, the contribution of MAT to the development of intestinal fibrosis in CD remains poorly understood [13]. The complexity of this relationship, balancing protective and fibrogenic roles, highlights a significant gap in our understanding of CD pathophysiology. Investigating the mechanisms by which MAT contributes to intestinal fibrosis is thus essential, offering potential insights for novel therapeutic strategies in managing CD.

In the complex interplay of CD pathophysiology, a key component emerging from MAT is its exosomes. These small extracellular vesicles, ranging from 30 to 150 nm in diameter, have gained recognition as fundamental players in intercellular communication [14]. By encapsulating a diverse array of biomolecules, including proteins, lipids, and nucleic acids, exosomes function beyond mere transporters; they are instrumental in modulating the behavior and function of recipient cells [15]. Facilitating a myriad of cellular interactions, from immune response regulation to cell proliferation and microenvironment alteration, exosomes, particularly those originating from MAT, may be pivotal in unraveling the molecular mechanisms of intestinal fibrosis in CD. Their ability to cross biological barriers and transport biomolecules positions them as vital contributors in CD's disease processes, offering new perspectives in understanding and potentially treating CD.

Exosome research extends its impact beyond CD. In oncology, they contribute to tumor progression and metastasis, carrying oncogenic factors [16]. As potential biomarkers and therapeutic targets, they offer new opportunities in cancer diagnosis and treatment [17]. In neurology, exosomes are involved in the propagation of pathogenic proteins in diseases like Alzheimer's, aiding our understanding of these conditions and guiding new treatments [18]. The multifaceted roles of exosomes across various pathologies underscore their potential as diagnostic and therapeutic tools, particularly in gastrointestinal diseases like CD.

Among the diverse biomolecules transported by exosomes, particular proteins play crucial roles in disease processes. One such protein that has garnered attention in this context is TINAGL1(Tubulointerstitial Nephritis Antigen-Like 1). TINAGL1 has garnered significant interest in cellular biology, particularly for its roles in cell adhesion and migration, processes central to tissue development and repair [19], [20], [21]. Research has established a connection between TINAGL1 and wound healing, as well as its involvement in the fibrotic process, through its influence on extracellular matrix remodeling [22], [23]. Although its role in general fibrosis has been explored, the specific function of TINAGL1 in the context of intestinal fibrosis, particularly in diseases such as Crohn's Disease, remains largely unexplored. This gap in knowledge highlights the need for further investigation into TINAGL1′s potential role in intestinal fibrosis, offering a promising avenue for new insights into the molecular mechanisms underlying this complex pathological condition.

Advances in CD research have yet to fully elucidate the molecular underpinnings of intestinal fibrosis, one of its critical complications. A particular area of ambiguity involves the role of TINAGL1 in MAT-derived exosomes, especially in their capacity to activate intestinal fibroblasts, a key process in fibrogenesis. This study aims to uncover these intricate molecular interactions, potentially identifying novel therapeutic targets for fibrosis in CD.

This investigation focuses on the effect of MAT-derived exosomes on intestinal fibrosis in CD, with an emphasis on their role in activating intestinal fibroblasts. Proteomic analyses have highlighted elevated TINAGL1 levels in these exosomes. We hypothesize that TINAGL1 interacts with SMAD4, a central player in the TGF-β signaling pathway, exacerbating fibrotic processes. Unraveling this pathway could offer new insights into CD's fibrogenesis and guide the development of targeted treatments.

## Materials and methods

### Key reagents and antibodies

Antibodies used in our experiments include TINAGL1 (ab69036), TSG101 (ab125011), CD9 (ab236630), TGF-β (ab215715), α-SMA (ab7817), Vimentin (ab92547), SMAD2 (ab40855), SMAD3 (ab40854), GAPDH (ab8245), and CTGF (ab209780) from Abcam (Cambridge, UK). FSP-1 (16105–1-AP), COL1A1 (67288–1-Ig), SMAD4 (10231–1-AP), COL3A1(22734–1-AP), COL5A1(67604–1-Ig), and COL6A1(17023–1-AP) were sourced from Proteintech (Wuhan, China). Alexa Fluor 488 (A-11001) and Alexa Fluor 594 (A-21207) were procured from Thermo Fisher Scientific (MA, USA).

Recombinant Human TINAGL1 Protein (9304-TU), Recombinant Mouse TINAGL1 Protein (9208-TI), and Recombinant Human Hexokinase 2 (HK2) Protein (8179-HK) were all obtained from R&D Systems, Minneapolis, MN, USA. Additionally, Recombinant Human COL6A1 (RG308562), COL6A2 (RG318704), COL6A3 (RKRC14888), and COL6A5 (REEA09600) were purchased from Abmart, Shanghai, China. Recombinant Human TGF-β1 (100–21) was obtained from PeproTech, Rocky Hill, NJ, USA. Chemicals including 2,4-Dinitrofluorobenzene (DNBS) (233226) were procured from Sigma-Aldrich, St. Louis, MO, USA. For RNA extraction, QIAzol (Cat. No. 79306) was sourced from Qiagen, Hilden, Germany, and the RevertAid cDNA Synthesis Kit (K1632) for RNA reverse transcription was acquired from Thermo Fisher Scientific, Waltham, MA, USA. SYBR Green (4367659) for quantitative PCR was procured from Life Technologies, Carlsbad, CA, USA. Additionally, for flow cytometry analysis, the Annexin V Apoptosis Detection Kit (40302ES), 2′,7′-dichlorofluorescein diacetate (DCFH-DA) (50101ES01), and the cell apoptosis inducer CCCP (40333ES60) were purchased from Yeasen Biotechnology Co., Ltd, Shanghai, China.

### Patient sample collection for the study

This study enrolled a total of 15 patients diagnosed with Crohn's Disease (CD), who underwent intestinal resection due to colonic strictures. Post-resection, mesenteric adipose tissues adjacent to the excised intestinal segments were isolated, and parts of the intestinal tissues were cryopreserved at −80 °C for further analyses. For the control group, colonic specimens and corresponding mesenteric adipose tissues were collected from 15 patients undergoing surgery for colorectal cancer. From these specimens, while the margins of the intestines were used for the isolation of intestinal fibroblasts, parts of these intestinal tissues were also cryopreserved at −80 °C for additional tests. All participants provided written informed consent, agreeing to the anonymous use of their samples for research purposes. This study adhered to the Declaration of Helsinki and received approval from the Ethics Committee of Wuhan Hospital.

### Methodology for the isolation and cultivation of primary colonic fibroblasts from colorectal cancer patients

Primary Colonic Fibroblasts (PCFs) were isolated using an established protocol [24]. In 2021, serosal samples from the macroscopically normal margins of resected ileum from colorectal cancer patients (n = 15) were collected at Wuhan Union Hospital. These tissue samples were carefully dissected into 0.5 mm sections and placed into culture dishes. The samples were then incubated in complete culture medium containing 10 % serum. Once the cells approached confluence, they were detached using TrypLE. The isolated cells were identified as colonic fibroblasts based on their characteristic morphology under optical microscopy, positive immunofluorescence staining for Fibroblast-Specific Protein-1 (FSP-1) and Vimentin, and negative staining for α-Smooth Muscle Actin (α-SMA). For experimental treatments, PCFs were exposed to 100 ng/ml recombinant murine TINAGL1 (rmTINAGL1) for 24 h, with PBS serving as the vehicle control to ensure the specificity of the treatment effects.

### Exosome isolation and identification from human and murine mesenteric adipose tissue

Mesenteric adipose tissues from both human (Crohn's Disease and colorectal cancer patients) and murine models were processed for exosome isolation, employing a method consistent across species, as detailed in previous literature [25]. The tissues were minced and subjected to enzymatic digestion using a collagenase solution. This was followed by centrifugation to obtain a supernatant rich in exosomes. Subsequent ultracentrifugation concentrated the exosomes, which were then resuspended in phosphate-buffered saline, filtered, and stored at −80 °C.

Characterization of the isolated exosomes was performed through a comprehensive approach. Transmission Electron Microscopy (TEM) using a JEM-1400 Plus (JEOL Ltd., Tokyo, Japan) visualized the exosomes and confirmed their characteristic morphology. Nanoparticle Tracking Analysis with a nanoFCM system (NanoFCM Co., Ltd., Nottingham, UK) was employed to determine the exosomes' concentration and size distribution, following the same referenced procedures.

### Animal models of Crohn's Disease in mice

#### Mice model for chronic intestinal inflammation and fibrosis induced by DNBS

Male C57BL/6 mice, aged 8 weeks, were obtained from HFK Bioscience, Beijing, China, for establishing a chronic intestinal inflammation and fibrosis model. The experiment divided the mice into two groups: a model group (MG) and a control group (NG), each comprising six mice. Chronic inflammation was induced using an established DNBS rectal administration protocol over six weeks [26], with doses escalating weekly: 2 mg, 2 mg, 4 mg, 4 mg, 6 mg, and 6 mg. Post-induction, the mice were euthanized, and their colons were harvested. The proximal sections of the colons were paraffin-embedded for histological analysis, including Hematoxylin and Eosin (H&E) and Masson's Trichrome staining. Concurrently, mesenteric adipose tissue was collected for exosome extraction.

#### Mice model for assessing the impact of exosome injection on fibrosis

In a separate set of experiments, four groups of male C57BL/6 mice (8 weeks old, n = 6 per group) were used: control group (CG), DNBS-induced intestinal inflammation group (DG), DNBS with NG-derived exosome injection group (DNEG), and DNBS with MG-derived exosome injection group (DMEG). Chronic intestinal inflammation was similarly induced by rectal DNBS administration for six weeks, following the aforementioned dosage schedule. Concurrently, exosomes were administered intravenously once a week. After the treatment period, mice were euthanized, and their colons were collected. The distal colons were partially embedded in paraffin for H&E, Masson's Trichrome, and immunofluorescence staining. The remaining tissues were preserved for histological sequencing, RNA, and protein analysis.

#### Mice model for assessing the impact of TINAGL1 on intestinal fibrosis

To explore the pro-fibrotic potential of TINAGL1, we employed a hydrogel composed of alginate (AL) and hyaluronic acid (HA) for its oral delivery to a mouse model. The hydrogel was formulated by combining sodium alginate (2.0 wt%) and hyaluronic acid (1.0 wt%), both sourced from Sigma-Aldrich, St. Louis, MO, USA. It was subsequently stabilized with a solution of calcium and zinc ions (Ca2^+^/Zn2^+^) to facilitate gelation under gentle conditions. Male C57BL/6 mice (8 weeks old, n = 6 per group) were used to evaluate the effects. The mice received dinitrobenzene sulfonic acid (DNBS) to induce intestinal inflammation. One group was treated with the AL/HA hydrogel containing TINAGL1 (0.5 mg/kg), while the control group received the hydrogel without TINAGL1. Treatments were administered by intragastric gavage (0.5 mL/mouse) twice weekly, alongside the DNBS regimen.

#### Histopathological and oxidative stress analysis of mouse proximal colon using H&E, DHE, and Masson staining

After euthanasia, a 0.5 cm segment of the proximal colon was excised from each mouse. The samples were first fixed in 4 % formaldehyde, adhering to standard histological procedures, and then embedded in paraffin. From these paraffin blocks, 5 μm thick sections of the proximal colon were prepared for subsequent staining.

Hematoxylin and Eosin (H&E) staining was performed on these sections. Hematoxylin stains cell nuclei blue, while eosin colors the cytoplasm and other tissue components in varying shades of pink. This staining technique facilitates a detailed assessment of the cellular structure and morphology, including inflammation, lymphocyte infiltration, crypt damage, and surface epithelium loss. At least two sections per sample were evaluated to determine the average score for these histopathological changes.

To assess oxidative stress, DHE staining was performed to detect superoxide anion production. DHE is oxidized by superoxides, converting it into a fluorescent compound that can be visualized under a fluorescence microscope. This method allows for the direct observation and quantification of reactive oxygen species in the tissue sections, providing insights into the oxidative stress status within the colon. At least two sections per sample were analyzed to ensure consistency and reliability in the measurements.

Masson's Trichrome staining was employed to highlight collagen fibers in blue, aiding in the identification and quantification of fibrotic changes. The extent of collagen deposition was semi-quantitatively analyzed using ImageJ software. Fibrosis severity was assessed on a scale from 0 (no significant fibrosis) to 5 (severe fibrosis). Additionally, the percentage of the section involved with fibrosis was scored as follows: 1 for 0–25 % involvement, 2 for 25–50 % involvement, 3 for 50–75 % involvement, and 4 for 75–100 % involvement. The final Intestinal Fibrosis Score was calculated by multiplying the fibrosis score by the percentage involvement score, a method based on established practices in previous studies [27]. At least two sections per sample were evaluated to determine the average score.

#### Visualization of mesenteric adipose tissue-derived exosome uptake in mouse colonic tissue via DiR labeling

To validate the uptake of mesenteric adipose tissue-derived exosomes by intestinal cells in mice, the exosomes were labeled with DiR, a lipophilic fluorescent dye from Thermo Fisher Scientific, Waltham, Massachusetts, USA. Following the labeling process, these DiR-tagged exosomes were intravenously injected into the mice. Approximately eight hours post-injection, the mice were euthanized, and their colons were isolated for analysis. The colonic tissues were then subjected to fluorescent staining to visualize the uptake of the labeled exosomes. This process allowed for the direct observation of exosome distribution and localization within the intestinal cells, employing fluorescence microscopy to detect the near-infrared signal of DiR, indicative of the exosomes' presence in the tissue.

### Isolation method for primary mouse colonic fibroblasts

Primary colonic fibroblasts were isolated from the colons of untreated mice. The procedure involved excising proximal colon sections, approximately 0.5 cm in length, from normal, healthy mice. These sections were carefully dissected into small fragments and then cultured in a medium optimized for fibroblast growth. Once the cells in culture approached confluence, they were enzymatically detached using TrypLE, a gentle detachment method preserving cell integrity. The successful isolation and purity of primary colonic fibroblasts were confirmed through morphological assessment using optical microscopy and specific immunofluorescence staining for fibroblast markers.

### Culture conditions for normal human colon epithelial cells

Normal human colon epithelial cells (CCD 841 CoN) were obtained from the American Type Culture Collection (ATCC, https://www.atcc.org). For culturing, the cells were maintained in Dulbecco’s Modified Eagle Medium (DMEM) supplemented with 10 % fetal bovine serum (FBS, cat. 10099; Gibco, Brooklyn, NY, USA) and 1 % antibiotic–antimycotic solution. The culture environment was controlled at 37 °C with a humidified atmosphere of 5 % (v/v) carbon dioxide.

### Rna-sequencing analysis in DNBS-induced mouse colonic tissue and TINAGL1-treated human fibroblasts

RNA sequencing was performed on colonic tissues from dinitrobenzene sulfonic acid (DNBS)-induced mice and primary human fibroblasts treated with TINAGL1 recombinant protein at OE Biotech Co., Ltd., Shanghai, China. The goal was to identify transcriptomic changes associated with intestinal fibrosis in the mouse model and to assess the impact of TINAGL1 treatment in human cells. The RNA extraction process from both mouse tissues and treated fibroblasts involved thorough quality and concentration assessments to ensure data reliability for high-throughput sequencing. Differential gene expression analysis was performed using a fold change threshold of an absolute value greater than 2, ensuring the identification of significant transcriptomic alterations.The subsequent differential gene expression analysis of these RNA samples involved advanced bioinformatics techniques using R packages.

### Proteomics sequencing methodology for mouse mesenteric adipose tissue exosomes and colonic tissues

The proteomic profiling of exosomes derived from mouse mesenteric adipose tissue was conducted using a label-free quantification approach at Echo Biotech Co., Ltd, Beijing, China. This method enabled the comprehensive and unbiased analysis of the exosomal proteome. Concurrently, colonic tissues were subjected to Data-Independent Acquisition (DIA) proteomic sequencing by SpecAlly Life Technology Co., Ltd, Wuhan, China, ensuring extensive coverage and accurate quantification of the tissue proteome. Differential protein expression analysis was performed using a fold change threshold of an absolute value greater than 1.5. For bioinformatics analysis, R language along with various specialized R packages were employed, facilitating robust statistical evaluation and data visualization.

### Western blotting for tissue and cell lysates

In our Western blotting protocol, both tissues and cell samples were lysed using RIPA buffer containing a 1 × concentration of protease and phosphatase inhibitor cocktail. The lysates underwent sonication, followed by centrifugation at 12,000g for 15 min at 4 °C to clarify the samples. The protein concentration in the supernatant was quantified using the bicinchoninic acid assay. For electrophoresis, the proteins were separated by SDS-PAGE and then transferred onto PVDF membranes. These membranes were blocked with skim milk for one hour to prevent non-specific binding and incubated with primary antibodies overnight at 4 °C on a shaker. Subsequent to this, the membranes were washed with TBST and incubated with secondary antibodies for an hour. The detection of protein bands was achieved using a chemiluminescence system, and the bands were quantified using Fiji software version 1.5. Throughout these procedures, GAPDH served as a loading control to ensure consistent protein loading and transfer.

### Quantitative real-time PCR (qPCR) analysis

Total RNA for quantitative real-time PCR (qPCR) was extracted using QIAzol reagent, and its concentration was determined using a NanoDrop spectrophotometer from Thermo Scientific, located in Waltham, Massachusetts, USA. The extracted RNA was then reverse-transcribed into cDNA for use as a template in qPCR. Gene expression levels were quantified using the LightCycler 480 system (Roche Diagnostics, Indianapolis, IN, USA), with relative gene expression normalized against GAPDH using the 2^-ΔΔCT^ method. Primer sequences for the qPCR were designed with Primer Premier 6.0 software (PREMIER Biosoft International, Palo Alto, USA). These primer sequences are listed in Table 1, providing reference for the targeted genes in the study.

**Table 1:** Primer Sequences for Quantitative Real-Time PCR (qPCR) Analysis.

Symbol	Forward Primer (5′ −> 3′)	Reverse Primer (5′ −> 3′)
*Acat2*	CCCAGACATCAGGGAGTAATGG	TCTATCGGATACTTCAGCGTCA
*Col1a1*	GCTCCTCTTAGGGGCCACT	ATTGGGGACCCTTAGGCCAT
*Tgfb*	CCACCTGCAAGACCATCGAC	CTGGCGAGCCTTAGTTTGGAC
Smad2	CGTCCATCTTGCCATTCACG	CTCAAGCTCATCTAATCGTCCTG
Smad3	TGGACGCAGGTTCTCCAAAC	CCGGCTCGCAGTAGGTAAC
Col1a1	GAGGGCCAAGACGAAGACATC	CAGATCACGTCATCGCACAAC
Ctgf	CAGCATGGACGTTCGTCTG	AACCACGGTTTGGTCCTTGG

### Immunofluorescence analysis of colon sections and treated cells

For immunofluorescence analysis of colon sections, the samples were first incubated with primary antibodies, all diluted to a 1:200 concentration, and kept overnight at 4 °C. This step was followed by an additional incubation period of 1 h at room temperature the next day. After incubation with the primary antibodies, the sections were washed three times with PBS and then stained with DAPI to highlight the nuclei. This staining was succeeded by a 1-hour incubation with fluorescent secondary antibodies and further PBS washes to remove any unbound antibodies.

In parallel, for cellular studies, cells were grown in confocal dishes and treated with either exosomes or recombinant TINAGL1 protein. Post-treatment, the cells were fixed using paraformaldehyde to preserve cellular structures for imaging. The fixed cells were then incubated with specific antibodies overnight, a crucial step for visualizing the targeted proteins. Imaging of both colon sections and cells was conducted using a Zeiss LSM800 confocal microscope, a sophisticated imaging system offering high-resolution and detailed visualization of the fluorescently labeled cells and tissues.

### Flow cytometry analysis for ROS and apoptosis

Human primary colonic fibroblasts and CCD 841 CoN cells were treated with mesenteric adipose-derived exosomes or recombinant TINAGL1 protein for 24 h. For ROS detection, fibroblasts were stained with 2′,7′–dichlorofluorescin diacetate (DCFH-DA), a dye that measures intracellular reactive oxygen species. Cells were incubated at 37 °C for 30 min, then washed and analyzed using a BD FACSAria II flow cytometer (BD, USA), with data processed via FlowJo software. As a positive control for ROS, cells were treated with 100 mM Rosup for 1.5 h prior to staining. Similarly, for apoptosis detection, CCD 841 CoN cells were stained with annexin V-fluorescein isothiocyanate (FITC) to detect early apoptotic events, then washed and analyzed using the same flow cytometer setup. For a positive control in apoptosis assays, cells were treated with 50 μM CCCP for 20 min to induce apoptosis. Results were quantified through FlowJo software, ensuring consistent measurement of both oxidative stress and apoptotic responses under the same experimental conditions.

### Co-immunoprecipitation assay for TINAGL1 and SMAD4 interaction

For the Co-Immunoprecipitation (Co-IP) assay, lysates from human primary colonic fibroblasts were prepared using a lysis buffer after treatment with TINAGL1 recombinant protein. TINAGL1 and SMAD4 were genetically tagged with FLAG and MYC, respectively, and the plasmids were sourced from Genechem (Shanghai, China). Antibodies specific to MYC and FLAG tags were added to the lysates and incubated overnight at 4 °C to form antibody-protein complexes. The next day, Protein A/G beads (Beyotime, Shanghai, China, Cat No: P2177M) were added to capture these complexes. After several hours, the beads were thoroughly washed to remove non-specifically bound proteins, the bound complexes were eluted, and the proteins were analyzed by SDS-PAGE followed by Western blotting, using antibodies against both FLAG and MYC tags to confirm the interaction between TINAGL1 and SMAD4, demonstrating their direct involvement in the signaling pathways associated with fibrogenesis.

### Wound healing assay for cell migration in primary colonic fibroblasts

In assessing cell migration, we conducted a wound healing assay on primary colonic fibroblasts undergoing various treatments. Cultured in 6-well plates with DMEM supplemented with 10 % FBS, the cells were grown to 90–100 % confluence. A linear wound was then meticulously created using a 200 µl pipette tip, followed by a PBS wash to remove detached cells. The culture was continued in serum-free DMEM to emphasize migration over proliferation. At 24 h post-treatment, we used an optical microscope to observe and photograph the wound closure, offering an efficient and direct method to evaluate the migration capacity of fibroblasts under different experimental conditions.

### Assessment of primary intestinal fibroblast proliferation using CCK8 assay

Primary human and mouse intestinal fibroblasts' proliferation was assessed using the Cell Counting Kit-8 (CCK8) assay, sourced from Selleck Chemicals, Houston, Texas, USA. Cells were seeded in 96-well plates and subjected to designated treatments. Following these treatments, CCK8 solution was added to each well and incubated for a predetermined period to facilitate color development. The absorbance at 450 nm was measured using a microplate reader, quantifying cell proliferation.

### Statistical methods and analysis criteria

In our study, statistical analyses were conducted to ensure rigorous evaluation of the data. All results are expressed as means ± standard deviations, providing a clear representation of the data's variability. To determine statistical significance, an unpaired two-tailed Student's *t*-test was used for comparisons between two groups. For comparisons involving multiple groups, one-way analysis of variance (ANOVA) was applied. In all instances, a *p*-value threshold of less than 0.05 was established to denote statistical significance.

#### Ethical approval and consent to participate

This research was meticulously conducted under the highest ethical standards. The study involving human participants received approval from the independent Ethics Committee of Wuhan Union Hospital (Approval No. 2020-S1097). Additionally, all animal experiments were conducted in strict accordance with the guidelines and were approved by the Animal Ethics Committee of Huazhong University of Science and Technology (Approval No. 2022-3383).

## Results

### Elucidating the impact of MAT-derived exosomes in DNBS-induced intestinal fibrosis in a mouse model of crohn's disease

Our research effectively developed a chronic intestinal inflammation model in mice using dinitrobenzene sulfonic acid (DNBS), closely resembling Crohn's Disease (CD) pathology. The mice were allocated into two distinct groups: the model group (MG), which received DNBS induction, and the normal control group (NG).

A comprehensive histological examination of colon tissues from these groups revealed markedly elevated levels of inflammation and fibrosis in the MG, in stark contrast to the NG. This was distinctly visualized in the Hematoxylin and Eosin (H&E) staining (Fig. S1A), as well as the Masson's trichrome staining (Fig. S1B), both illustrating the extent of intestinal inflammation and fibrosis in the MG. Further quantitative assessments included Histological and Fibrosis scoring (Fig. S1C, D), which substantiated the visual observations with statistical data. Additionally, measurements of colon lengths (Fig. S1E) demonstrated a significant reduction in the MG, further corroborating the establishment of the fibrotic model. Gene expression analysis, focusing on fibrosis-related markers such as Acta2, Col1a1, and Tgfb, revealed their upregulated expression in the MG (Fig. S1F).

**Fig. 1:**
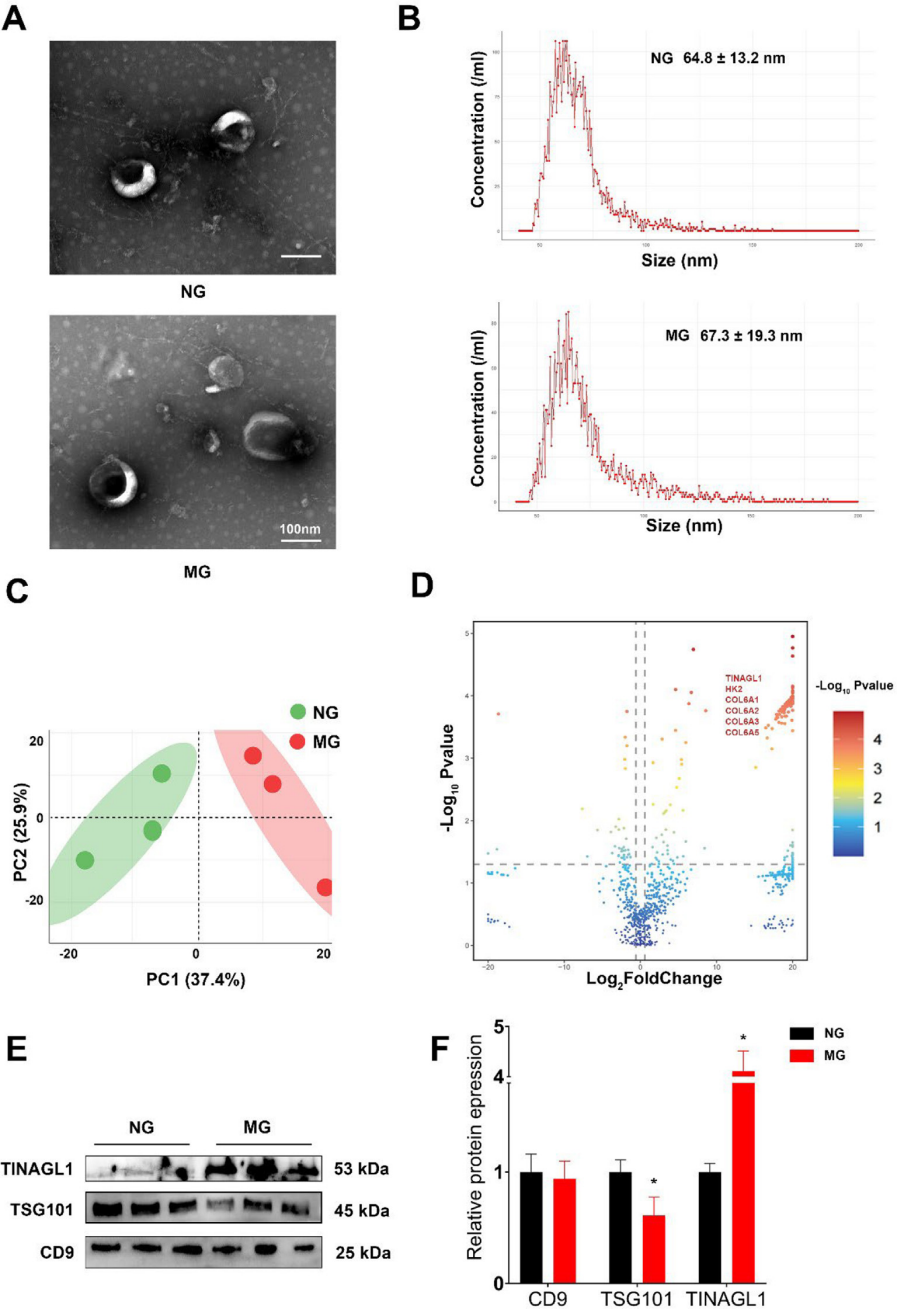
**Exosomal Characterization and Differential Protein Expression in DNBS-Induced Mouse Model** (A) Transmission Electron Microscopy (TEM) images reveal typical exosomal morphology in exosomes from mesenteric adipose tissue (MAT) in both model group (MG) and normal controls (NG) (Scale bar: 100 nm). (B) Nanoflow Cytometry analysis indicates a subtle variation in median diameter of exosomes between MG (approximately 67.3 ± 19.3 nm) and NG (approximately 64.8 ± 13.2 nm). (C) Principal Component Analysis (PCA) plot illustrates distinct proteomic profiles, demonstrating clear differentiation between MG and NG. (D) Volcano Plot highlights the upregulation of proteins like TINAGL1, HK2, COL6A1, COL6A2, COL6A3, and COL6A5 in MG, suggesting their potential role in fibrogenesis. (E) Western blot analysis confirms the presence of exosomal markers TSG101 and CD9 in both groups, with a marked increase in TINAGL1 levels in MG. (F) Quantitative analysis of Western blot data, showing significantly elevated expression of TINAGL1 in MG. (n = 3–6; Statistical significance determined using an unpaired two-tailed Student's *t*-test, *p < 0.05).

RNA sequencing of colonic tissues from the MG was undertaken to delve deeper into the molecular alterations. The gene expression volcano plot (Fig. S2A) and the circular heatmap of differential gene expression (Fig. S2B) consistently displayed a coherent pattern of gene expression changes within the group. Enrichment analyses of differentially expressed genes through Gene Ontology (GO) (Fig. S2C-E) and Kyoto Encyclopedia of Genes and Genomes (KEGG) pathways (Fig. S2F) further emphasized the aggregation of genes in pathways directly associated with fibrogenesis. These combined findings from histological, molecular, and bioinformatics analyses strongly indicate the successful establishment of a DNBS-induced model of intestinal fibrosis in mice.

**Fig. 2:**
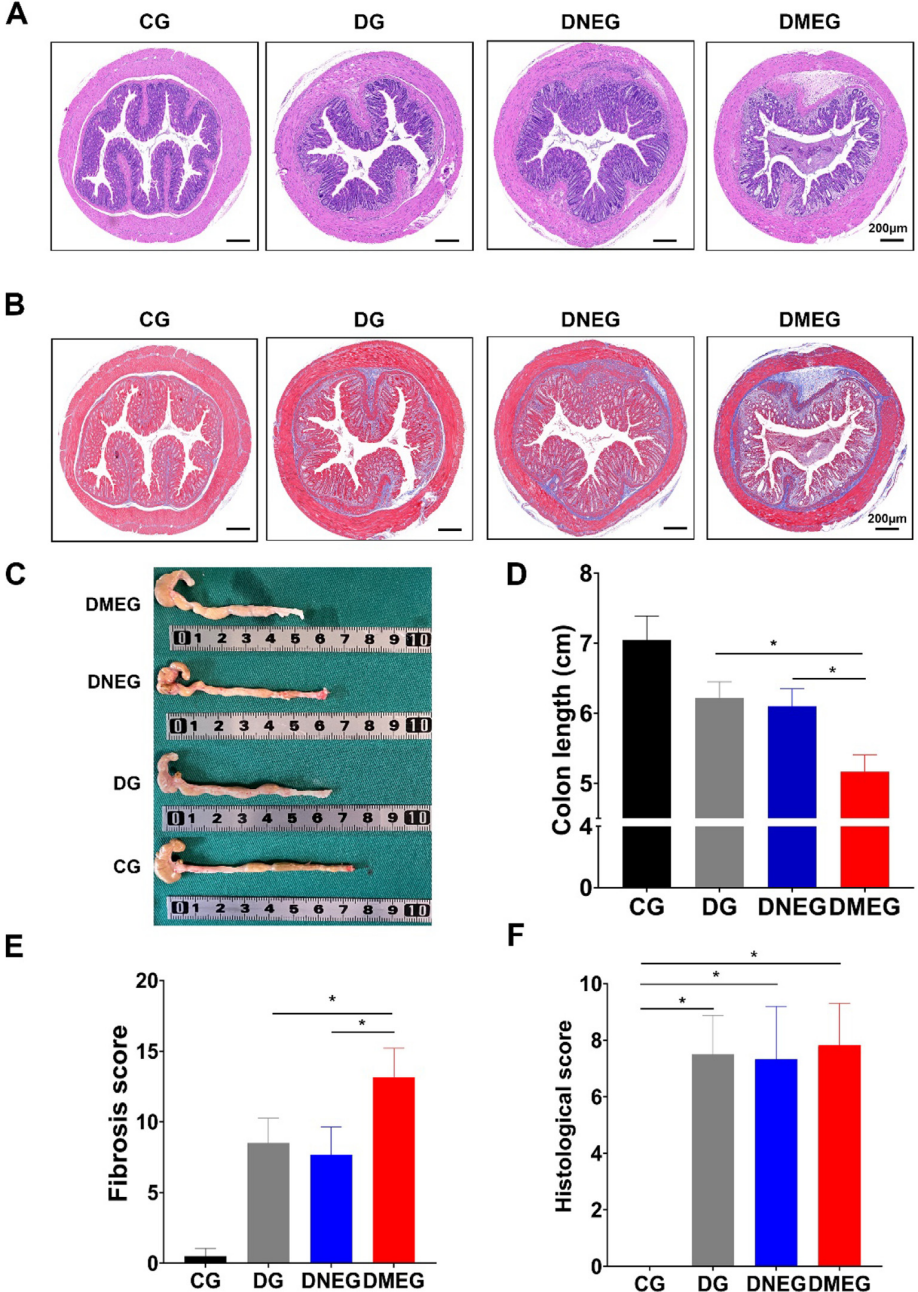
**Analysis of Exosome Uptake by Colonic Fibroblasts and Its Impact on Fibrosis in DNBS-Induced Mouse Models** (A) Hematoxylin and Eosin (H&E) staining of colon sections in DNBS-treated groups (DNBS-Induced Group [DG], DNBS with Normal Group Exosomes [DNEG], DNBS with Model Group Exosomes [DMEG]) and the Control Group (CG). Scale bar: 200 µm. (B) Masson’s Trichrome staining of colon tissue sections from CG, DG, DNEG, and DMEG groups. Scale bar: 200 µm. (C) Photographs of extracted mouse colons from all groups. (D-F) Statistical analyses of colon length, Histological score, and Fibrosis score. The DMEG group exhibits shorter colons and higher Fibrosis scores compared to other groups, with no significant difference in Histological scores among DMEG, DNEG, and DG groups (n = 3–6; Statistical significance determined by one-way ANOVA, *p < 0.05).

Following euthanasia, mesenteric adipose tissue (MAT) was isolated from both groups to extract exosomes. Transmission electron microscopy (TEM) revealed the typical exosomal structure in samples from both groups (Fig. 1A). Nanoflow cytometry analysis indicated that the median diameter of exosomes from the MG was approximately 67.3 ± 19.3 nm, while in the NG, it was about 64.8 ± 13.2 nm (Fig. 1B). Proteomic profiling of the exosomes demonstrated distinct clustering between the MG and NG in the principal component analysis (PCA) plot (Fig. 1. C).

A detailed exploration of differential protein expression using a volcano plot identified a notable increase in proteins including TINAGL1, HK2, COL6A1, COL6A2, COL6A3, and COL6A5 in the MG (Fig. 1D), previously implicated in fibrogenic processes. The circle heat map of differential proteins (Fig. S3A) and the GO enrichment analysis results, covering Biological Processes, Cellular Components, and Molecular Functions (Fig. S3B-D), further elucidated the proteomic variations. KEGG pathway enrichment analysis (Fig. S3E) and classification of enriched pathways (Fig. S3F) provided additional insights into the biological significance of these protein changes.

**Fig. 3:**
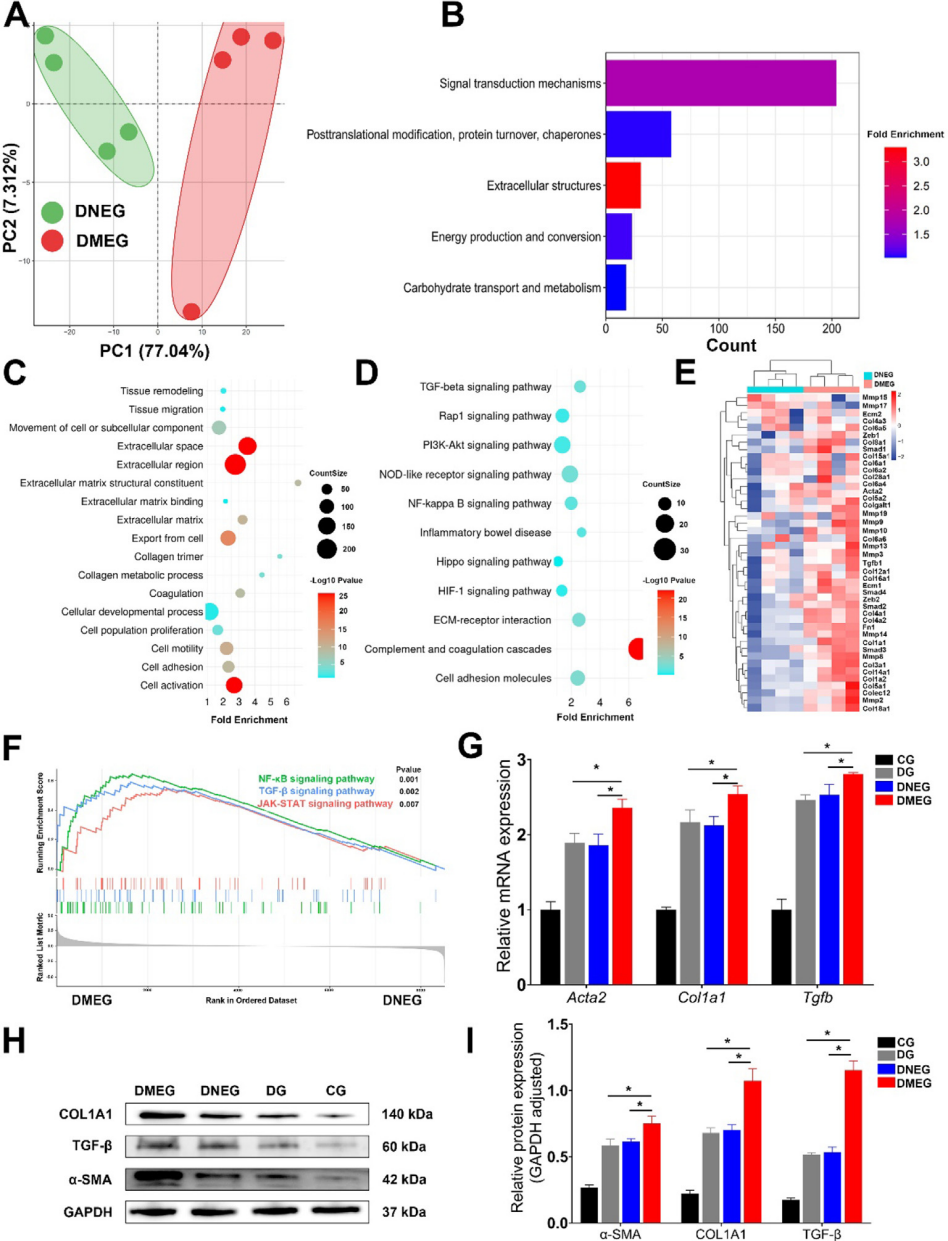
**Proteomic and Molecular Analysis of Intestinal Fibrosis in DNBS-Induced Mouse Models with MAT-Derived Exosome Treatment** (A) Principal Component Analysis (PCA) depicting distinct proteomic profiles in DMEG (DNBS with Model Group Exosomes) and DNEG (DNBS with Normal Group Exosomes) groups. (B) Clusters of Orthologous Groups (COG) enrichment analysis of differential proteins. (C) Gene Ontology (GO) enrichment analysis of differential proteins, revealing significant enrichment in fibrosis-related terms such as Collagen trimer, Collagen metabolic process, Extracellular space, and Extracellular matrix. (D) Kyoto Encyclopedia of Genes and Genomes (KEGG) pathway enrichment analysis, indicating differential proteins enriched in pathways like TGF-β signaling pathway, ECM-receptor interaction, Complement and coagulation cascades, as well as inflammation-related pathways including NF-κB and NOD-like receptor signaling pathways, suggesting cross-talk between inflammation and fibrosis. (E) Heatmap analysis demonstrates elevated expression of collagen family proteins, fibronectin (FN1), and matrix metalloproteinases (MMPs) in the DMEG group compared to the DNEG group. (F) Gene Set Enrichment Analysis (GSEA) highlighting significant activation of NF-κB, TGF-β, and JAK-STAT signaling pathways in the DMEG group compared to DNEG, underscoring the fibrotic process augmentation. (G) PCR analysis of colonic tissues from all groups, showing increased expression of fibrosis markers *Acta2*, *Tgfb*, and *Col1a1* in the DMEG group compared to DNEG and DG groups, with no significant difference between DNEG and DG, GAPDH was used as the internal control. (H and I) Western blot analysis showing increased levels of fibrosis markers α-SMA, TGF-β, and COL1A1 in the DMEG group compared to DNEG, with GAPDH serving as an internal control. Statistical analysis was performed using one-way ANOVA (n = 3–6; *p < 0.05 indicates statistical significance).

To determine the effects of various proteins on fibroblast activity, we treated human primary colonic fibroblasts with recombinant proteins TINAGL1, HK2, COL6A1, COL6A2, COL6A3, and COL6A5. Our results indicated that TINAGL1 specifically upregulated key fibrogenic markers including COL1A1, α-SMA, and TGF-β (Fig. S4A-B). Furthermore, treatment with recombinant human TGF-β led to an increase in TINAGL1 expression (Fig. S4C-D), highlighting the crucial role of TINAGL1 in fibroblast activation and the fibrogenesis process. While the role of TINAGL1 in wound healing and general fibrogenesis has been recognized, its specific impact on intestinal fibrosis, particularly within the context of Crohn's Disease, remains largely uncharted.

**Fig. 4:**
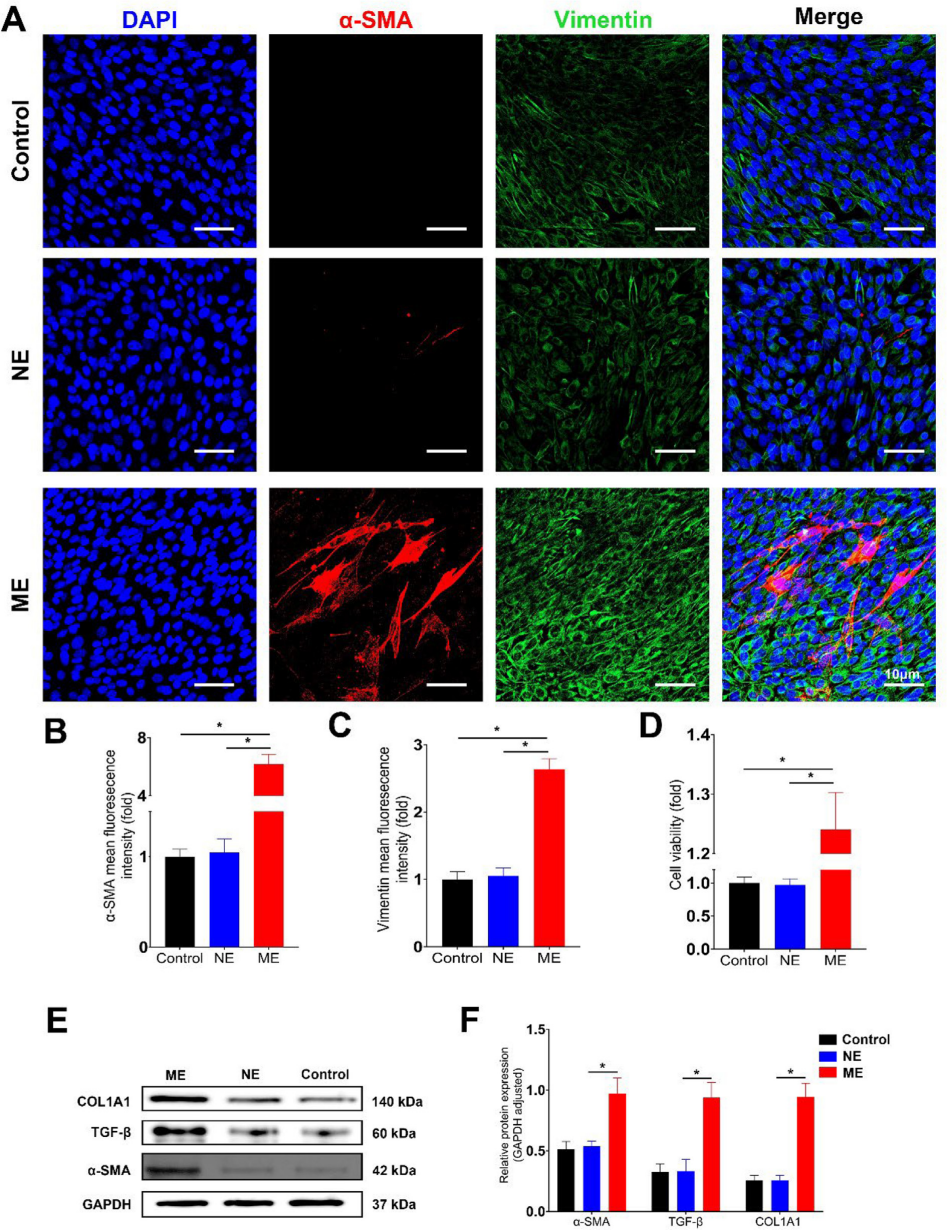
**Assessment of MAT-Derived Exosome Effects on Fibroblast Activation in Mouse Colonic Cells** (A) Immunofluorescence staining of isolated mouse colonic fibroblasts treated with exosomes. Control group received no treatment, NE group was treated with Normal Group exosomes, and ME group with Model Group exosomes. Staining for α-SMA (red) and Vimentin (green) indicates fibroblast activation(Scale bar: 10 µm). (B and C) Quantitative analysis of fluorescence intensity shows significantly higher α-SMA and Vimentin expression in the ME group compared to NE, suggesting enhanced fibroblast activation. (D) Cell viability assessed by CCK8 assay demonstrates increased viability in the ME group compared to NE. (E and F) Western blot analysis reveals differential protein expression across the groups. Notably, in the ME group (treated with Model Group Exosomes), there are elevated levels of α-SMA, TGF-β, and COL1A1 compared to the NE group (treated with Normal Group Exosomes) and the untreated Control group. GAPDH serves as an internal control for normalization across all groups. Quantitative analysis confirms the upregulation of these fibroblast activation markers in the ME group. Statistical analysis was performed using one-way ANOVA (n = 3–6; *p < 0.05 indicates statistical significance). (For interpretation of the references to color in this figure legend, the reader is referred to the web version of this article.)

Western blot analysis confirmed the presence of the exosomal markers TSG101 and CD9 in both groups. Crucially, the levels of TINAGL1 were significantly higher in the MG compared to the NG (Fig. 1E and F). This observation highlights TINAGL1′s significant impact on intestinal fibrosis, emphasizing its role in exacerbating fibrotic processes, particularly in Crohn's Disease. Given the elevated levels of TINAGL1 observed, further studies are needed to understand its precise mechanisms and implications for fibrosis progression, which could inform potential strategies to mitigate its effects.

### Evaluating the effects of MAT-derived exosomes on fibrosis progression in a DNBS-induced mouse model of Crohn's Disease

In our study, mice were categorized into four groups: Control Group (CG), DNBS-Induced Group (DG), DNBS with Normal Group Exosomes (DNEG), and DNBS with Model Group Exosomes (DMEG). We explored the interaction of exosomes with colonic fibroblasts by labeling intravenously injected exosomes with DiR. Immunofluorescence analysis demonstrated that fibroblasts, identified with FSP-1 in the colon, absorbed DiR-labeled exosomes in both DMEG and DNEG groups (Fig. S5A), with no marked differences in the absorption levels (Fig. S5B, C). Additionally, the results indicated that the number of fibroblasts was increased in the DMEG group compared to the DNEG group, suggesting that the exosomes from the model group may enhance fibroblast proliferation or recruitment in the context of DNBS-induced intestinal fibrosis.

**Fig. 5:**
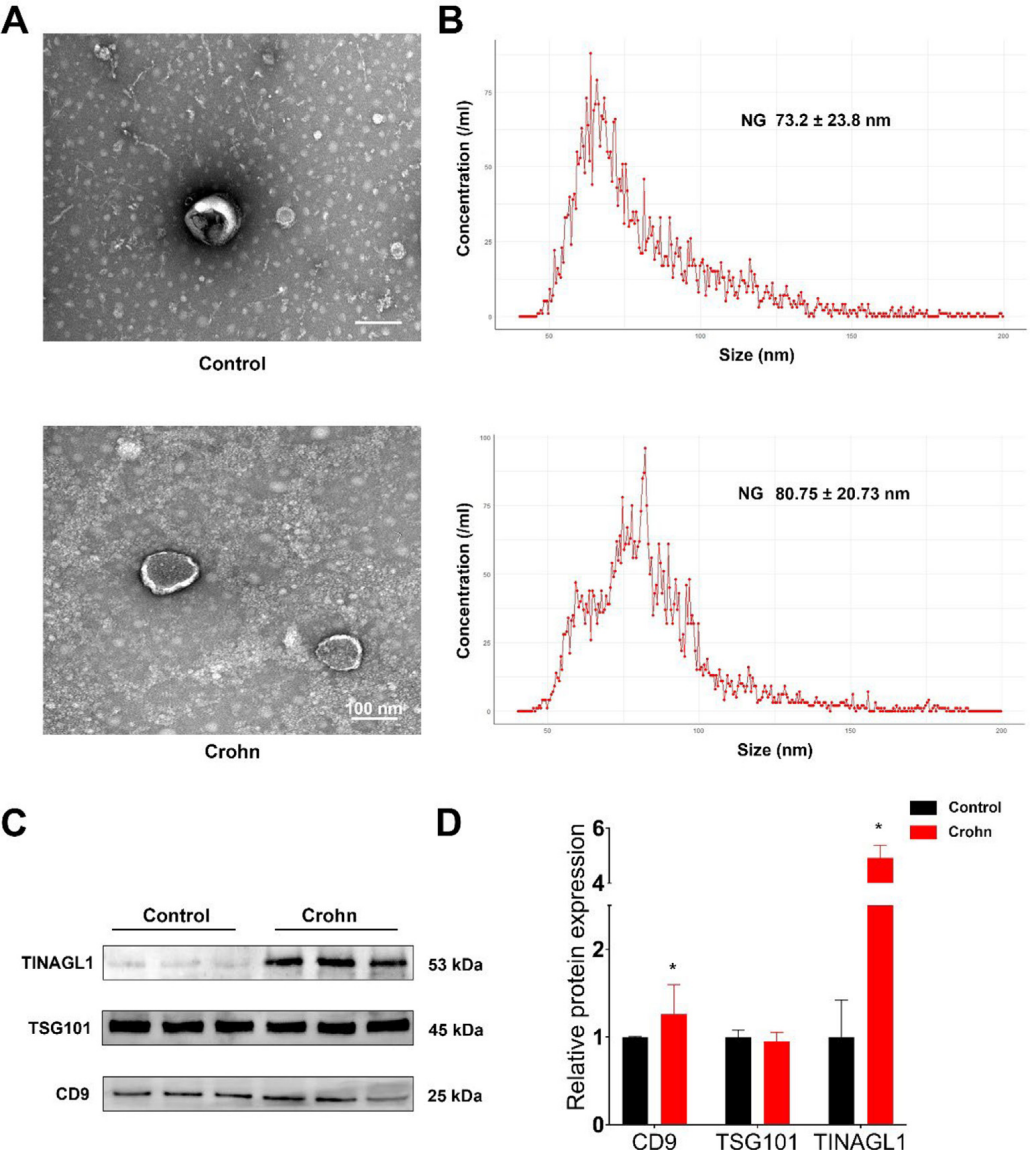
**Characterization of Mesenteric Adipose Tissue-Derived Exosomes from Crohn's Disease Patients and Control Group** (A) Transmission Electron Microscopy (TEM) images displaying the ultrastructure of exosomes isolated from mesenteric adipose tissue of Crohn's Disease patients (Crohn group) and cancer patients (Control group). The images showcase the characteristic vesicular shapes typical of exosomes(Scale bar: 100 nm). (B) Nanoflow cytometry analysis illustrating the size distribution of exosomes. The median diameter of exosomes from the Crohn group was approximately 80.75 ± 20.73 nm, compared to about 33.2 ± 23.8 nm in the Control group, revealing a variation in exosome size between the two groups. (C) Western Blot Analysis of Exosomal Proteins: The Western blot provided a visual representation of the protein content, displaying bands for exosomal markers TSG101 and CD9, as well as TINAGL1, in both Control and Crohn group exosomes. (D) Quantitative Evaluation of Protein Levels: This panel represents the quantitative analysis of the Western blot, showing the relative expression levels of TSG101, CD9, and TINAGL1. The analysis revealed a notably higher expression of TINAGL1 in the Crohn group compared to the Control group (n = 3–6; Statistical significance determined by using an unpaired two-tailed Student's *t*-test, *p < 0.05).

Hematoxylin and Eosin (H&E) staining of the colon sections (Fig. 2A) revealed increased intestinal inflammation in the DNEG, DMEG, and DG groups compared to the CG group. Masson’s Trichrome staining further highlighted that the DMEG group exhibited significantly more pronounced fibrosis compared to the other groups (Fig. 2B). Subsequent post-mortem examinations of the colon illustrated these inflammatory and fibrotic changes more comprehensively (Fig. 2C). Statistical analyses of colon length and fibrosis scores further supported these findings, showing shorter colons (Fig. 2D) and higher fibrosis scores (Fig. 2E) in the DMEG group, indicative of an enhanced fibrotic response. Interestingly, despite these findings, no significant differences in Histological scores among the DG, DNEG, and DMEG groups were observed, suggesting that MAT-derived exosomes may not significantly affect the degree of intestinal inflammation.

Proteomic sequencing of the DMEG and DNEG groups displayed distinct clustering (Fig. 3A). COG enrichment analysis identified a significant enrichment in Extracellular structures, suggesting a link with fibrogenesis (Fig. 3B). Further, GO and KEGG enrichment analyses (Fig. 3C, D) emphasized pathways closely related to fibrosis, such as Collagen trimer and TGF-β signaling pathway.

Heatmap visualization illustrated elevated expression of collagen family proteins, fibronectin (FN1), and matrix metalloproteinases (MMPs) in the DMEG group compared to the DNEG group, emphasizing the role of these proteins in enhancing fibrotic activity (Fig. 3E). GSEA analysis further confirmed the activation of key fibrosis-related pathways in the DMEG group, including the TGF-β signaling pathway, suggesting that exosomes from the MG group potentiate fibrosis (Fig. 3F). TGF-β is well-known for its role in promoting fibrosis by stimulating collagen production and facilitating the transition of fibroblasts to myofibroblasts. The TGF-β/Smad signaling pathway is crucial in this process: TGF-β binds to its type II serine/threonine kinase receptor (TGFBR II), which activates the type I receptor (TGFBR I). TGFBR I then phosphorylates Smad2 and Smad3, which form a complex with Smad4 [28]. This complex enters the nucleus to regulate the transcription of genes that drive fibrosis, including those in intestinal fibrosis [29].

To further validate the biochemical impact of these findings, PCR and Western blot analyses corroborated the upregulation of fibrosis markers α-SMA, TGF-β, and COL1A1 in the DMEG group relative to the DNEG and DG groups (Fig. 3G-I). These findings indicate that exosomes derived from the MG group significantly promote intestinal fibrosis, likely through the modulation of key fibrotic pathways and the enhancement of fibroblast activity.

Further, we isolated colonic fibroblasts from CG mice and divided them into three groups: untreated (Control), treated with NG-derived exosomes (NE), and treated with MG-derived exosomes (ME). Immunofluorescence staining for α-SMA (red) and Vimentin (green) showed increased fluorescence intensity in ME compared to NE (Fig. 4A). Quantitative analysis confirmed stronger α-SMA and Vimentin signals in ME (Fig. 4, C). CCK8 assay indicated higher cell viability in ME (Fig. 4D). Western blot results showed increased expression of α-SMA, TGF-β, and COL1A1 in ME compared to NE (Fig. 4E, F), indicating a significant impact of MG-derived exosomes on fibroblast activation and fibrosis.

The comprehensive data from our mouse model experiments elucidate the significant role of MAT-derived exosomes in modulating fibrosis in a DNBS-induced model of Crohn's Disease. Our findings demonstrate that exosomes from the Model Group (MG) notably enhance fibrotic processes, as evidenced by increased expression of key fibrotic markers and alterations in relevant signaling pathways. The observed effects on colonic fibroblasts, both in terms of exosome uptake and subsequent fibrotic response, underscore the potential of these exosomes as modulators of fibrosis. This sets a strong foundation for our subsequent investigations into the interactions between human colonic cells and MAT-derived exosomes, furthering our understanding of the cellular mechanisms underpinning fibrosis in Crohn's Disease.

### Impact of mesenteric adipose tissue-derived exosomes from Crohn's Disease patients on fibroblast activation and fibrogenesis

Following our insights from the mouse model, we extended our investigation to human mesenteric adipose tissue (MAT) derived from Crohn's Disease patients and a control group, termed as Crohn and Control groups, respectively. Transmission electron microscopy (TEM) revealed distinct exosomal structures in both groups (Fig. 5A). Nanoflow cytometry indicated a significant difference in median exosome diameter between Crohn (80.75 ± 20.73 nm) and Control (33.2 ± 23.8 nm) groups (Fig. 5B). Western blot analysis confirmed the presence of exosomal markers TSG101 and CD9 in both groups, with an increase in TINAGL1 in the Crohn group (Fig. 5C and D).

Despite established connections between inflammation and enhanced fibrosis, our study demonstrated that injections of model group exosomes did not exacerbate intestinal inflammation in mice. Considering the important role reactive oxygen species (ROS) play in the progression of inflammatory bowel disease (IBD) [30], we evaluated the effect of these exosomes on ROS levels in colonic and fibroblast cells.

**Fig. 6:**
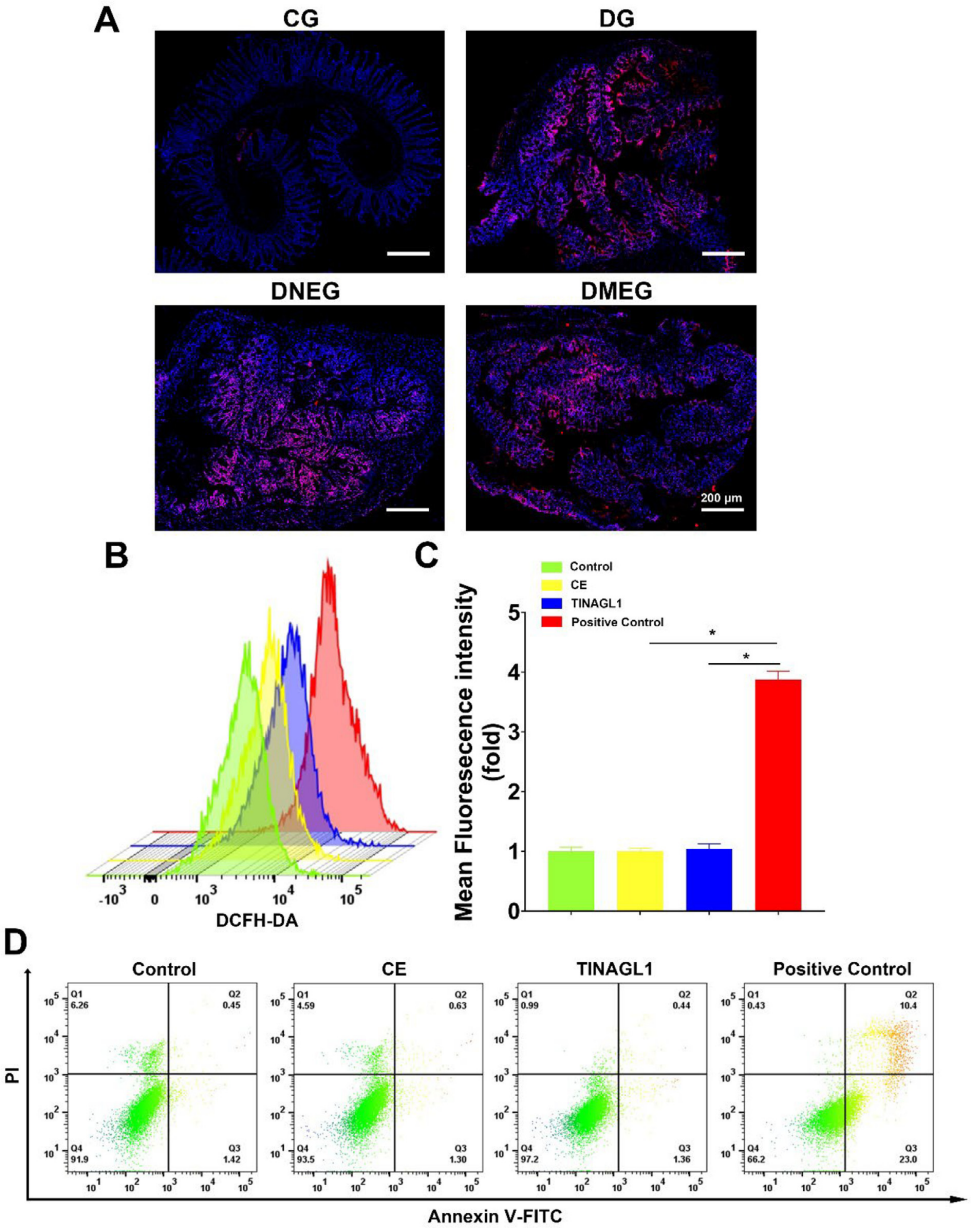
**Impact of Mesenteric Adipose-Derived Exosomes on ROS Levels and Apoptosis** (A) Dihydroethidium (DHE) staining images displaying reactive oxygen species (ROS) levels in colonic tissues from four mouse groups: DNBS with Normal Group Exosomes (DNEG), DNBS with Model Group Exosomes (DMEG), DNBS-induced Group (DG), and Control Group (CG), Scale bar: 200 µm. (B) Flow cytometric analysis of ROS in primary human colonic fibroblasts using DCFH-DA staining, showing responses in untreated control, cells treated with Crohn group exosomes (CE), cells treated with recombinant TINAGL1 protein, and a positive control. (C) Quantitative summary of flow cytometry results for ROS levels, providing a statistical comparison among treatment groups. (D) Apoptosis assessment in CCD841 CoN human colon epithelial cells via flow cytometry, detailing the apoptotic profiles in untreated control, cells treated with Crohn group exosomes (CE), and cells treated with recombinant TINAGL1 protein, alongside a positive control. (n = 6; statistically analyzed using one-way ANOVA, with *p < 0.05 indicating significance.).

Dihydroethidium (DHE) staining revealed no apparent differences in ROS production among the DMEG, DNEG, and DG groups (Fig. 6A). Additionally, in vitro experiments with human primary colonic fibroblasts treated with Crohn group exosomes or recombinant TINAGL1 protein, assessed via flow cytometry using DCFH-DA staining, also showed no increase in ROS (Fig. 6B-C). Moreover, given the crucial role of intestinal epithelial cells in maintaining gut barrier integrity [31], we conducted apoptosis assays on normal human colon epithelial cells (CCD 841 CoN) treated with either Crohn group exosomes or TINAGL1 protein. These assays showed no significant increase in cell apoptosis (Fig. 6D).

These results suggest that the fibrogenic action of Crohn group exosomes in our models does not proceed via an increase in epithelial cell apoptosis or fibroblast ROS production, pointing to other potential mechanisms by which these exosomes may contribute to intestinal fibrosis. This finding steers our research towards uncovering these alternative pathways, enhancing our understanding of how Crohn's disease-associated fibrosis develops.

**Fig. 7:**
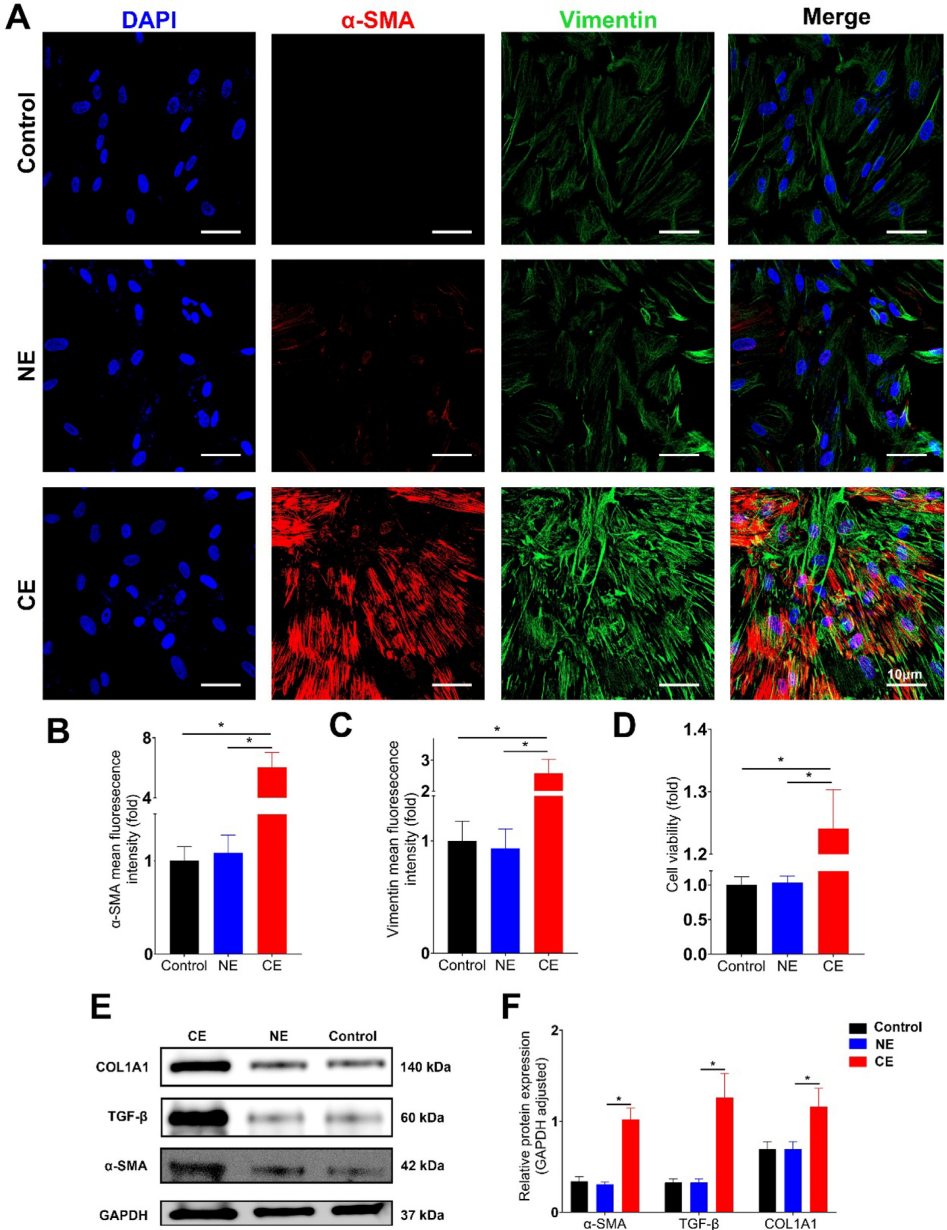
**Effect of Crohn's Disease Patient-Derived Exosomes on Human Intestinal Fibroblast Activation** (A) Immunofluorescence staining of primary human intestinal fibroblasts. Fibroblasts were divided into three groups: untreated control, treated with exosomes from the control group (NE), and treated with exosomes from Crohn's Disease patients (CE). Staining indicates α-SMA (red) and Vimentin (green), markers for fibroblast activation(Scale bar: 10 µm). (B and C) Quantitative analysis of fluorescence intensity. α-SMA and Vimentin expression levels are significantly higher in the CE group compared to NE, indicating a more pronounced activation of fibroblasts upon treatment with Crohn's Disease patient-derived exosomes. (D) Cell viability assay using CCK8. The CE group shows increased cell viability compared to NE. (E and F) Western blot analysis of fibroblast activation markers. The CE group exhibits increased expression of α-SMA, TGF-β, and COL1A1 compared to NE, with GAPDH serving as an internal control. Quantitative analysis of protein expression levels, confirming the upregulation of fibrosis-related markers in the CE group. (n = 3–6; Statistical significance determined by one-way ANOVA, *p < 0.05). (For interpretation of the references to color in this figure legend, the reader is referred to the web version of this article.)

To further elucidate the mechanisms behind the fibrogenic effects of Crohn group exosomes, we investigated their impact on primary human intestinal fibroblasts. These cells were divided into three groups: untreated Control, treated with Control group exosomes (NE), and treated with Crohn group exosomes (CE). Immunofluorescence staining revealed pronounced α-SMA (red) and Vimentin (green) fluorescence in the CE group (Fig. 7A), with significantly higher intensity compared to the NE group (Fig. 7B, C). Cell viability assays indicated enhanced viability in the CE group (Fig. 7D). Western blot analysis showed increased α-SMA, TGF-β, and COL1A1 levels in the CE group relative to NE (Fig. 7E, F). Additionally, a wound healing assay was conducted to assess cell migration capacity. Photographic evidence showed accelerated wound closure in fibroblasts treated with CE compared to those treated with NE (Fig. S6A). Quantitative analysis confirmed significantly faster wound healing in the CE group, highlighting the impact of Crohn group exosomes on fibroblast migration and wound closure (Fig. S6B).

**Fig. 8:**
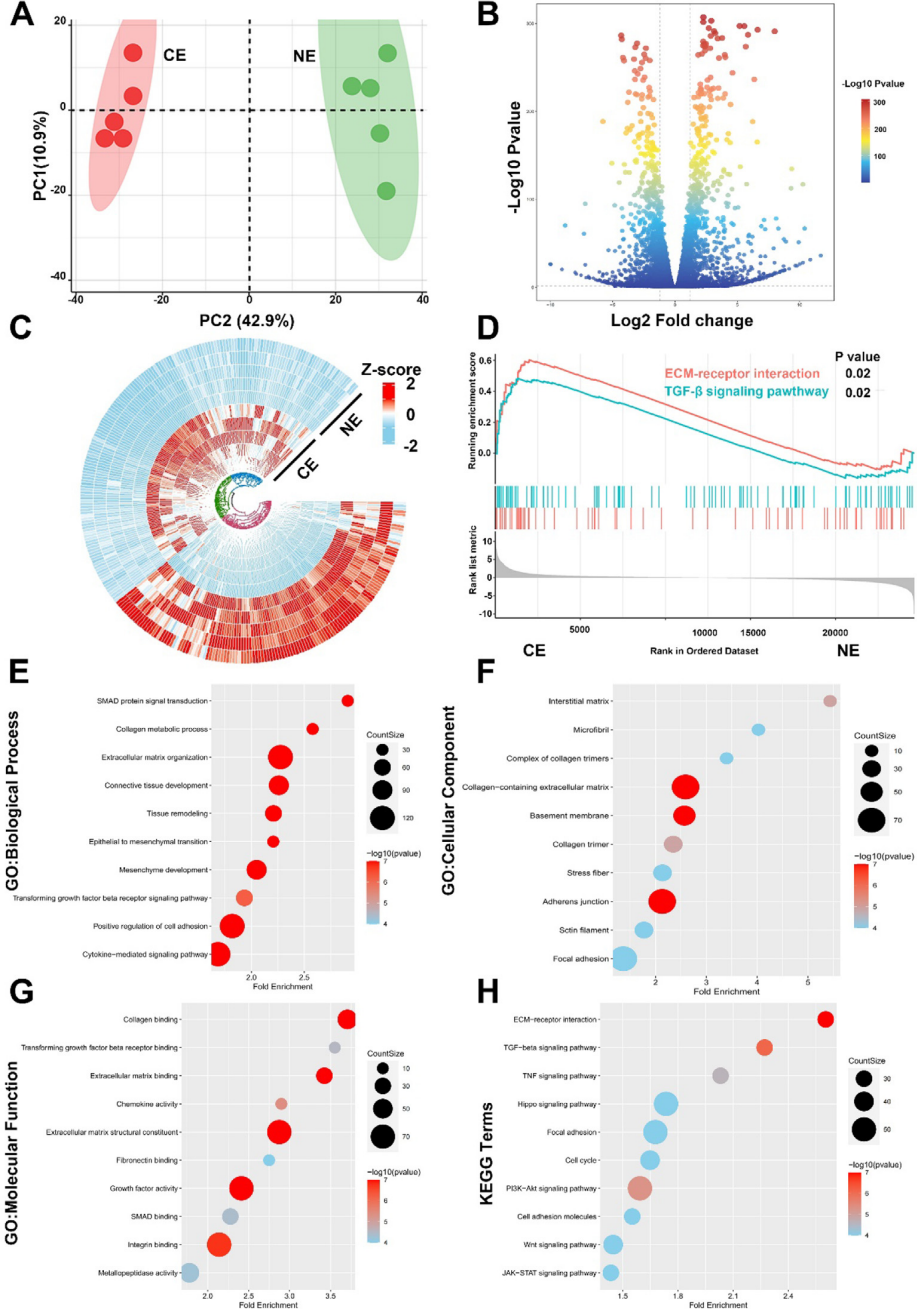
**RNA-Sequencing Analysis of Human Intestinal Fibroblasts Treated with Crohn's Disease and Control Group Exosomes** (A) Principal Component Analysis (PCA) illustrating distinct gene expression profiles in fibroblasts treated with exosomes from the Crohn's Disease group (CE) compared to those treated with the control group exosomes (NE). (B) Volcano plot representing differential gene expression between the CE and NE groups, highlighting the genomic response to the exosomal treatments. (C) Circular heatmap depicting the differential gene expression patterns, emphasizing the genomic alterations induced by the distinct treatments. (D) Gene Set Enrichment Analysis (GSEA) indicating significant pathway activations in the CE group, reflective of the fibrogenic influence of Crohn's Disease patient-derived exosomes. (E-G) Gene Ontology (GO) enrichment analysis showing significant enrichment in fibrosis-related terms across Biological Processes (E), Cellular Components (F), and Molecular Functions (G). (H) Kyoto Encyclopedia of Genes and Genomes (KEGG) pathway analysis revealing enrichment in key fibrosis-associated pathways, underscoring the influence of Crohn's Disease patient-derived exosomes on fibrogenesis. Experimental replicates n = 5.

To further investigate the molecular basis of these changes, we performed RNA sequencing of the NE and CE groups, which exhibited distinct gene expression profiles (Fig. 8A). Differential gene analysis identified 3528 genes (Fig. 8B), with a circular heatmap showing high within-group consistency (Fig. 8C). GSEA analysis indicated significant activation of ECM-receptor interaction and TGF-β signaling pathways in the CE group (Fig. 8D). GO enrichment analysis pointed to fibrosis-related terms (Fig. 8E-G), and KEGG pathway analysis highlighted involvement in ECM-receptor interaction and TGF-beta signaling pathways (Fig. 8H). These results confirm the fibrogenic role of Crohn group exosomes in activating fibroblasts, supporting their significant contribution to the progression of fibrosis.

Our research into human MAT-derived exosomes from Crohn's Disease patients builds on our previous mouse model findings, uncovering their significant role in fibrogenesis. The alteration in fibroblast behavior, gene expression, and activation of fibrosis-related pathways in response to Crohn's exosomes underscore their critical role in the fibrotic processes of Crohn's Disease. This study highlights the intricate relationship between MAT-derived exosomes and intestinal fibrosis, suggesting these exosomes as key modulators in the fibrotic landscape of Crohn's Disease.

### Exploring TINAGL1′s effect on fibroblast activation and TGF-β pathway engagement

**Fig. 9:**
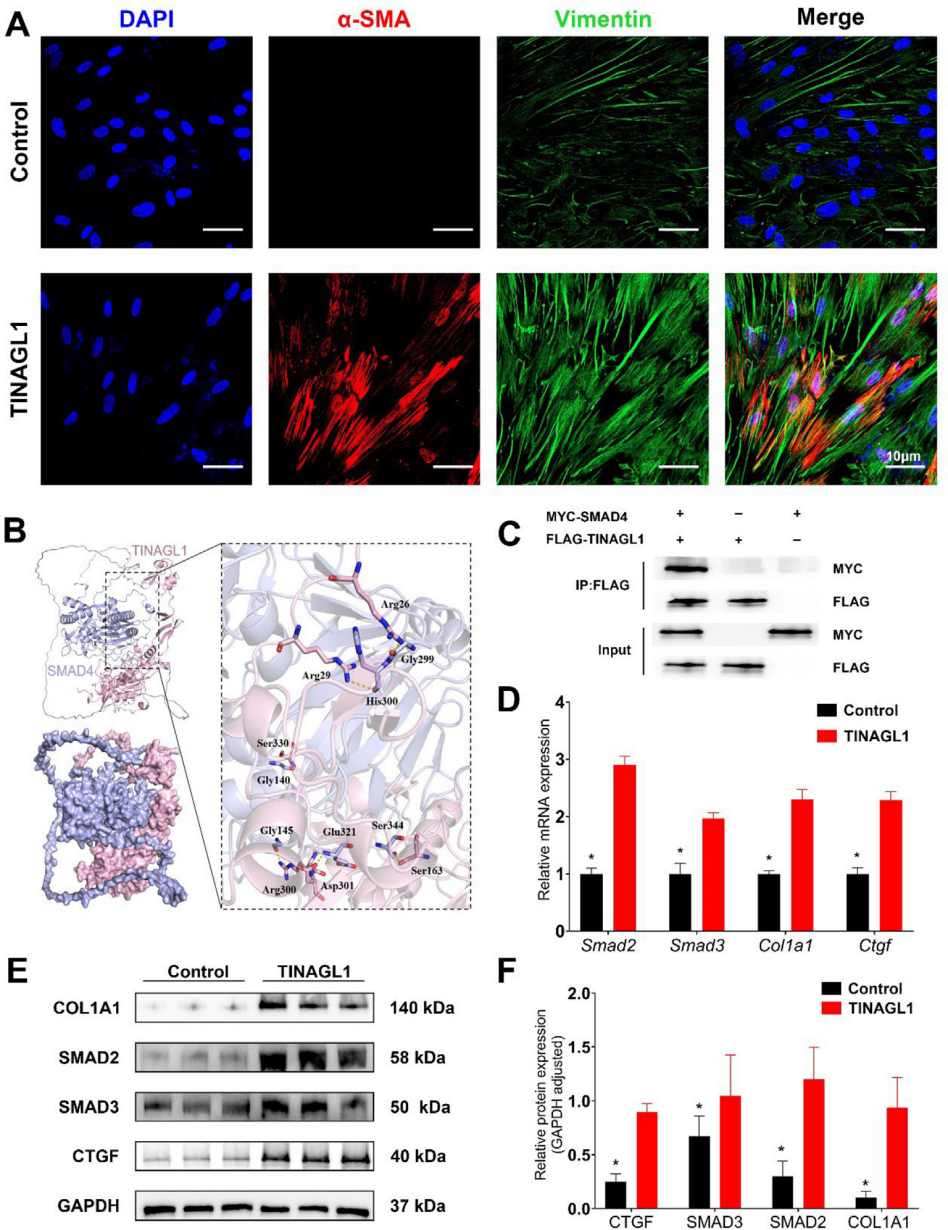
**Role of TINAGL1 in Modulating Fibroblast Activation and TGF-β Signaling** (A) Immunofluorescence staining of human primary intestinal fibroblasts treated with recombinant TINAGL1 protein, showing α-SMA (red) and Vimentin (green) staining. (B) Protein docking analysis (Discovery Studio software) suggesting a potential interaction between TINAGL1 and SMAD4. (C) Co-immunoprecipitation (Co-IP) assays following co-transfection of FLAG-tagged TINAGL1 and MYC-tagged SMAD4 into human intestinal fibroblasts. The interaction was validated using anti-FLAG and anti-MYC antibodies. (D) PCR analysis of gene expression in fibroblasts treated with TINAGL1 compared to control, with GAPDH serving as an internal reference. (E) Western blot analysis of SMAD2, SMAD3, and COL1A1 proteins in TINAGL1-treated fibroblasts, with GAPDH as an internal control. (F) Quantitative analysis of Western blot results. (n = 3–6; Statistical significance determined by one-way ANOVA, *p < 0.05). (For interpretation of the references to color in this figure legend, the reader is referred to the web version of this article.)

Primary human intestinal fibroblasts were treated with recombinant TINAGL1 protein to investigate its role in fibroblast activation and TGF-β pathway modulation. Immunofluorescence staining revealed that cells treated with TINAGL1 exhibited significantly increased α-SMA (red) and Vimentin (green) fluorescence intensity compared to the control group, indicating enhanced fibroblast activation (Fig. 9A). Photographic evidence demonstrated more rapid wound closure in fibroblasts treated with recombinant TINAGL1 protein compared to the control group (Fig. S7A). Quantitative analysis further confirmed significantly accelerated wound healing in the TINAGL1-treated fibroblasts, underscoring the protein's effect on enhancing fibroblast migration and wound closure capabilities (Fig. S7B).

To investigate the potential interaction of TINAGL1 with key transcription factors in the TGF-β signaling pathway, including R-SMADs, Co-SMADs, and I-SMADs, protein docking simulations and Co-Immunoprecipitation (CoIP) assays were conducted. Molecular docking simulations using Discovery Studio software revealed a substantive interaction between TINAGL1 and SMAD4 proteins, highlighted by the formation of hydrogen bonds, as evidenced by yellow dashed lines in the docking visualization. Specifically, amino acids Gly140, Gly145, Gly299, His300, Glu321, and Ser344 of SMAD4 were observed to form hydrogen bonds with Ser330, Arg300, Arg26, Arg29, Asp301, and Ser163 of TINAGL1, respectively. This detailed interaction mapping underscores the potential functional significance of the TINAGL1-SMAD4 complex in modulating cellular pathways. (Fig. 9B). Subsequent Co-Immunoprecipitation (CoIP) assays validated the interaction between TINAGL1 and SMAD4. Using antibodies specific to FLAG and MYC tags, which were genetically added to TINAGL1 and SMAD4 respectively, we confirmed their direct interaction (Fig. 9C).

To align the fibrogenic activity of TINAGL1 with the collagen types predominantly found in Crohn's Disease pathology, Western blot analyses were performed. These showed a marked increase in COL1A1 protein expression in both the narrowed sections of colons from Crohn's Disease patients and in human primary colonic fibroblasts treated with TINAGL1 (Fig. S8A-D). This finding supports the hypothesis that TINAGL1 plays a crucial role in the fibrogenesis observed in Crohn's Disease.

Gene expression changes following treatment with recombinant TINAGL1 protein were assessed using PCR. We observed significant upregulation in the expression of Smad2, Smad3, Col1a1, and Ctgf in TINAGL1-treated cells compared to controls (Fig. 9D). These findings were supported by Western blot analyses, which also indicated increased levels of these proteins in the TINAGL1-treated group (Fig. 9E, F), suggesting activation of the TGF-β signaling pathway.

To further investigate the in vivo effects of TINAGL1, we utilized an alginate (AL) and hyaluronic acid (HA) hydrogel for oral delivery of recombinant TINAGL1 protein to a mouse model subjected to intestinal inflammation induced by DNBS. Mice were divided into two groups: one received AL/HA hydrogel containing TINAGL1, referred to as the DGH_T group, and the control group received hydrogel without TINAGL1, termed the DGH group. Notably, the DGH_T group exhibited significantly higher levels of fibrosis compared to the DGH group (Fig. S9A-D).

These results collectively suggest that increased expression of TINAGL1 in mesenteric adipose tissue-derived exosomes may contribute to intestinal fibrogenesis by facilitating the activation of the TGF-β/SMAD4 signaling pathway, highlighting a potential mechanistic pathway for fibrosis enhancement in intestinal tissues.

## Discussion

**Fig. 10:**
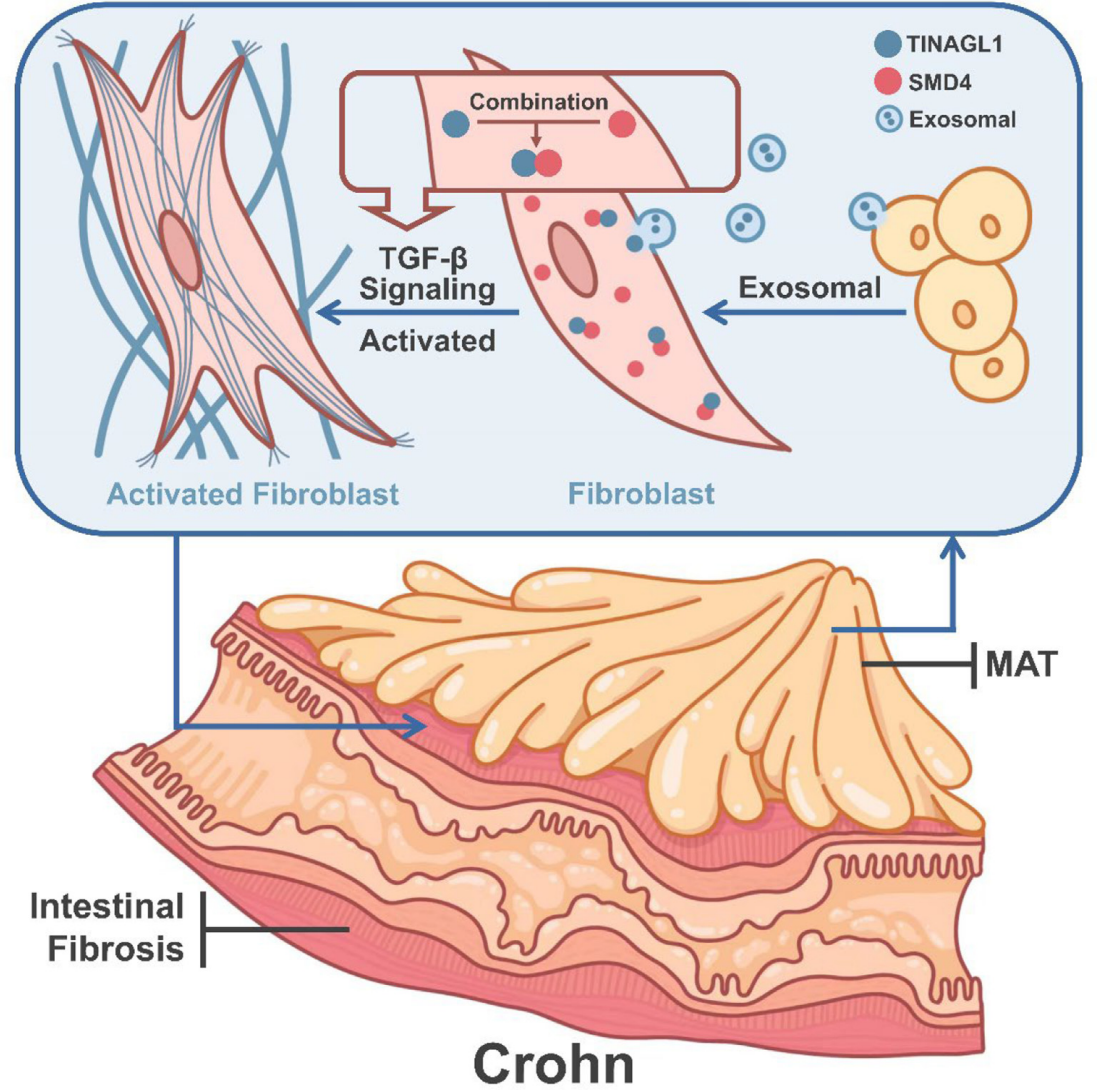
**Schematic Illustration of the Study Mechanism in Crohn's Disease** Elevated TINAGL1 expression in mesenteric adipose tissue (MAT) from Crohn's Disease patients enhances TGF-β signaling through its interaction with SMAD4, contributing to colonic fibroblast activation and exacerbating intestinal fibrosis. This schematic underscores the crucial role of TINAGL1 in Crohn's Disease-associated fibrogenesis, demonstrating how molecular alterations in MAT-derived exosomes influence the pathogenesis of intestinal fibrosis.

Our study unveils pivotal insights into the role of TINAGL1 in the pathogenesis of intestinal fibrosis, particularly focusing on Crohn's Disease. We discovered that TINAGL1, enriched in mesenteric adipose tissue-derived exosomes from Crohn's Disease patients, significantly influences fibrogenesis (Fig. 10). This was evidenced by the enhanced activation of fibroblasts, demonstrated by the increased expression of α-SMA and Vimentin (Fig. 9A), markers indicative of a fibroblast-to-myofibroblast transition [32]. This transition is a key event in fibrogenesis, where myofibroblasts, characterized by their high contractility and prolific extracellular matrix production, are central to the development of fibrotic tissue [33], [34].

Further supporting our findings, we observed an upregulation of fibrosis markers such as SMAD2, SMAD3, and COL1A1 (Fig. 9D-F), linking TINAGL1′s effect to molecular changes associated with fibrogenesis. The interaction between TINAGL1 and SMAD4, uncovered through protein docking and Co-Immunoprecipitation assays (Fig. 9B, C), unveils a novel mechanism of TINAGL1 action. This interaction suggests that TINAGL1 could augment the TGF-β signaling cascade, pivotal in the transcriptional regulation of fibrosis-related genes.

Significantly, our research has illuminated the role of TINAGL1 in modulating primary fibroblast behavior, a crucial factor in intestinal fibrosis. By utilizing primary fibroblasts isolated from humans and mice, we have shown that Crohn's Disease patient-derived mesenteric adipose tissue exosomes can activate these fibroblasts (Fig. 4, Fig. 7). This activation leads to a transformative state where fibroblasts evolve into myofibroblasts, key drivers of fibrosis through excessive extracellular matrix deposition. Thus, our study not only deepens the understanding of fibrotic processes in Crohn's Disease but also identifies a potential therapeutic target in TINAGL1.

The importance of these findings lies in their potential application in Crohn's Disease management. The fibrotic complications in Crohn's Disease present a substantial challenge [35], and our research suggests that targeting TINAGL1 could offer a new approach to treatment. By revealing the direct interaction between TINAGL1 and SMAD4, our study highlights how this specific interaction influences the TGF-β signaling pathway. The binding of TINAGL1 to SMAD4, a key transcription factor in the pathway, suggests a mechanistic route through which TINAGL1 modulates fibroblast behavior and contributes to the progression of fibrosis.

In the dynamic landscape of gastrointestinal disease research, our findings about TINAGL1′s role in Crohn's Disease offer a fresh perspective, particularly in understanding intestinal fibrosis. MAT in Crohn's Disease has been a topic of dual interpretation in the scientific community. On one hand, some researchers argue that MAT acts as a protective barrier, preventing the spread of inflammation and thereby mitigating more severe inflammatory responses [36], [13]. On the other hand, there is growing evidence, which our study supports, that MAT in Crohn's Disease may exacerbate intestinal fibrosis [12], [37], [38].

Additionally, it is important to note that intestinal epithelial cells are central regulators of gut immune homeostasis, and their excessive apoptosis can lead to increased intestinal permeability [31], [39]. This in turn triggers chronic intestinal inflammation, exacerbating intestinal fibrosis. In our experiments involving the MAT-derived exosomes and recombinant TINAGL1 protein intervention on the CCD841 CoN cell line, we observed no increase in apoptosis (Fig. 6D). Furthermore, ROS are closely associated with levels of inflammation and play a significant role in the activation of fibroblasts [30], [40]. However, our interventions with MAT-derived exosomes and recombinant TINAGL1 protein on primary human colonic fibroblasts did not result in an increase in ROS (Fig. 6B and C). This suggests that the fibrogenic effects of these exosomes and TINAGL1 may operate through mechanisms independent of epithelial apoptosis and ROS production.

TINAGL1, previously established as closely linked to wound healing and implicated in the metastasis of breast cancer [23], [41], [42], has now been identified in our research as significantly upregulated in the MAT of Crohn's Disease patients. Our findings reveal that TINAGL1 promotes the expression of COL1A1 in fibroblasts, aligning with the increased type I collagen observed in the narrowed intestinal sections of Crohn's patients (Fig. S8). Further, TINAGL1 expression was found to increase in fibroblasts treated with TGF-β, underscoring its pivotal role in fibroblast activation (Fig. S4C and D). This marks a pioneering step in associating TINAGL1 with the aggravation of intestinal fibrosis in Crohn's Disease. Our study not only identifies the increased expression of TINAGL1 but also unravels its potential mechanism of action. We demonstrate that TINAGL1 may interact with SMAD4, enhancing the activity of the TGF-β signaling pathway, a crucial element in fibrotic processes [43], [44].

Furthermore, our research underscores the critical role of exosomes in cellular communication within the intestinal environment. While previous studies have confirmed that MAT contributes to intestinal fibrosis in Crohn's Disease [37], [45], our investigation is the first to conduct a proteomic analysis of these exosomes and their specific protein expression profiles. The innovative aspect of our work lies in the detailed exploration of these exosomal contents and their functional implications.

By treating primary human colonic fibroblasts with exosomes derived from Crohn's Disease patient MAT, and utilizing RNA sequencing, we provided concrete evidence of TGF-β pathway activation (Fig. 7). This finding is crucial as it underscores the pivotal role of colonic fibroblasts in the progression of intestinal fibrosis. These fibroblasts, responding to signals such as those from the TGF-β pathway, produce an excessive amount of extracellular matrix (ECM), which is a key factor leading to fibrosis [46], [47]. Our findings not only corroborate the involvement of MAT in this process but also shed light on the mechanistic pathway, highlighting the fibroblasts' production of ECM as a central element. Consequently, our study bridges a critical gap in the current understanding of Crohn's Disease pathophysiology and opens new avenues for targeted therapeutic interventions, particularly those aimed at modulating fibroblast activity and ECM production.

In summary, our work situates itself at the forefront of current research by providing novel insights into the dual role of MAT in Crohn's Disease, the significant impact of TINAGL1 in this context, and the intricate molecular communication mediated by exosomes, which together drive the fibrotic processes in this debilitating condition.

In our investigation of TINAGL1′s role in intestinal fibrosis, particularly in Crohn's Disease, we acknowledge inherent limitations. Primary human fibroblasts and murine models, while informative, may not fully mirror human fibrosis complexity.

Our study has primarily focused on the impact of exosomes on colonic fibroblasts, which play a significant role in fibrogenesis. However, other cell types also contribute to intestinal fibrosis, warranting broader investigation in future studies. Additionally, the gut microbiome plays a crucial role in the pathogenesis of IBD, and emerging research suggests that nanozymes can influence gut microbial composition [48]. Exploring the interaction between mesenteric adipose tissue-derived exosomes and the gut microbiome represents a significant area for future research. Furthermore, these exosomes contain various proteins that, while not the focus of our current study, may be relevant to fibrogenesis, suggesting additional unexplored pathways and interactions that could help elucidate the mechanisms of intestinal fibrosis more comprehensively.

Future research should include in vivo studies to validate our findings in a more comprehensive biological context. Investigating the role of TINAGL1 in other fibrotic diseases could broaden the understanding of its mechanisms. Clinical trials focusing on TINAGL1 modulation could also be pivotal in translating these findings into therapeutic interventions for fibrosis in Crohn's Disease and beyond.

Our study represents a significant advancement in the field of fibrosis research, particularly within the context of Crohn's Disease. By elucidating the role of TINAGL1, predominantly found in mesenteric adipose tissue-derived exosomes of Crohn's Disease patients, we have identified a novel pathway contributing to intestinal fibrosis. The interaction between TINAGL1 and SMAD4, and its subsequent influence on the TGF-β signaling pathway, highlights a previously uncharted mechanism in fibrogenesis.

The novelty of our findings lies in the detailed characterization of TINAGL1′s role and the mechanistic insights provided into how it exacerbates fibrosis. This research not only deepens our understanding of the complex role of MAT in Crohn's Disease pathophysiology but also opens up potential avenues for therapeutic intervention. Targeting TINAGL1 or its pathways could offer new strategies for managing and treating the fibrotic complications frequently observed in Crohn's Disease.Furthermore, the implications of our study extend beyond Crohn's Disease. The methodologies employed and findings obtained can serve as a model for investigating fibrosis in other diseases, potentially leading to broader applications in fibrotic disease management. In conclusion, our research contributes substantially to the growing body of knowledge in fibrosis and Crohn's Disease, paving the way for future investigations and innovative clinical approaches to combat these challenging conditions.

## Conclusions

Our comprehensive study unveils a novel and significant aspect of CD pathophysiology, highlighting the critical role of TINAGL1-enriched exosomes derived from MAT in promoting intestinal fibrosis. By delineating the molecular mechanisms underlying this process, we have identified the interaction between TINAGL1 and the SMAD4 protein, elucidating its crucial involvement in the TGF-β signaling pathway, a central pathway in fibrogenesis.

This research advances our understanding of the complex interplay between MAT and intestinal fibrosis in CD. The increased expression of TINAGL1 in MAT-derived exosomes and its subsequent impact on fibroblast activation underscores a key molecular link in the exacerbation of fibrosis. Our findings not only enhance the current knowledge in the field but also provide a potential therapeutic target, opening new avenues for the management and treatment of fibrotic complications in CD.

Moreover, the methodologies and insights gained from this study have broader implications, offering a template for exploring fibrosis mechanisms in other gastrointestinal and systemic diseases. The implications of our research extend beyond CD, suggesting that similar mechanisms might be at play in other fibrotic conditions, thereby contributing to a wider understanding of fibrosis and its treatment.

In conclusion, our study represents a significant step forward in fibrosis research, particularly within the realm of Crohn's Disease. It lays the groundwork for future investigations and potential clinical applications, aiming to alleviate the burden of this debilitating aspect of CD and improve patient outcomes.

## Credit author statement

C.Y.D. conducted the primary experiments, led the manuscript writing, and performed data analysis, covering the roles of Investigation and Writing original draft. L.J.R., Z.X.P., L.S., C.Y.Y., F.X.Y., and L.J.M. managed and maintained the mouse models throughout the study, fulfilling the roles of Resources and Data curation. Z.L.R. conceptualized and designed the study, oversaw the research process, and secured funding, corresponding to the roles of Conceptualization, Supervision, and Funding acquisition. Additionally, Z.L.R. was instrumental in revising and finalizing the manuscript.

## Consent for Publication

All identifiable data from individuals in this study are included with explicit consent. Participants provided written informed consent for the publication of their case details and any other personal or clinical information. The consent forms are retained by the authors' institution and can be reviewed by the Editor-in-Chief of this journal upon request.

## Data availability

The data that support the findings of this study are available from the corresponding author upon reasonable request. To ensure comprehensive accessibility and transparency, the mass spectrometry proteomics data have been deposited in the ProteomeXchange Consortium, accessible via the iProX partner repository, under the dataset identifier PXD047519. Furthermore, the RNA-sequencing data associated with this study are available in the NCBI Sequence Read Archive (SRA) database. Two distinct datasets have been uploaded, with the accession numbers PRJNA10485560 and PRJNA1049332.

## Source of funding

This study was supported by the National Key R&D Program of China under Grant Numbers 2023YFC2507300 and 2018YFC0114600. Additional support was provided by the National Natural Science Foundation of China, Grant Numbers 82170547 and 81873558.

## Ethical Approval and Consent to participate

This research was meticulously conducted under the highest ethical standards. The study involving human participants received approval from the independent Ethics Committee of Wuhan Union Hospital (Approval No. 2020-S1097). Additionally, all animal experiments were conducted in strict accordance with the guidelines and were approved by the Animal Ethics Committee of Huazhong University of Science and Technology (Approval No. 2022-3383).

## Declaration of competing interest

The authors declare that they have no known competing financial interests or personal relationships that could have appeared to influence the work reported in this paper.
