# Supplementary material for: Mesenteric adipose-derived exosomal TINAGL1 enhances intestinal fibrosis in Crohn's Disease via SMAD4
Source: J Adv Res. 2024 May 13;70:139–58. doi: 10.1016/j.jare.2024.05.016 (PMC11976418; doi:10.1016/j.jare.2024.05.016)
Supplement: Supplementary Data 1 [file mmc1.docx]

SUPPLEMENTARY FIGURES

**Figure S1**

**
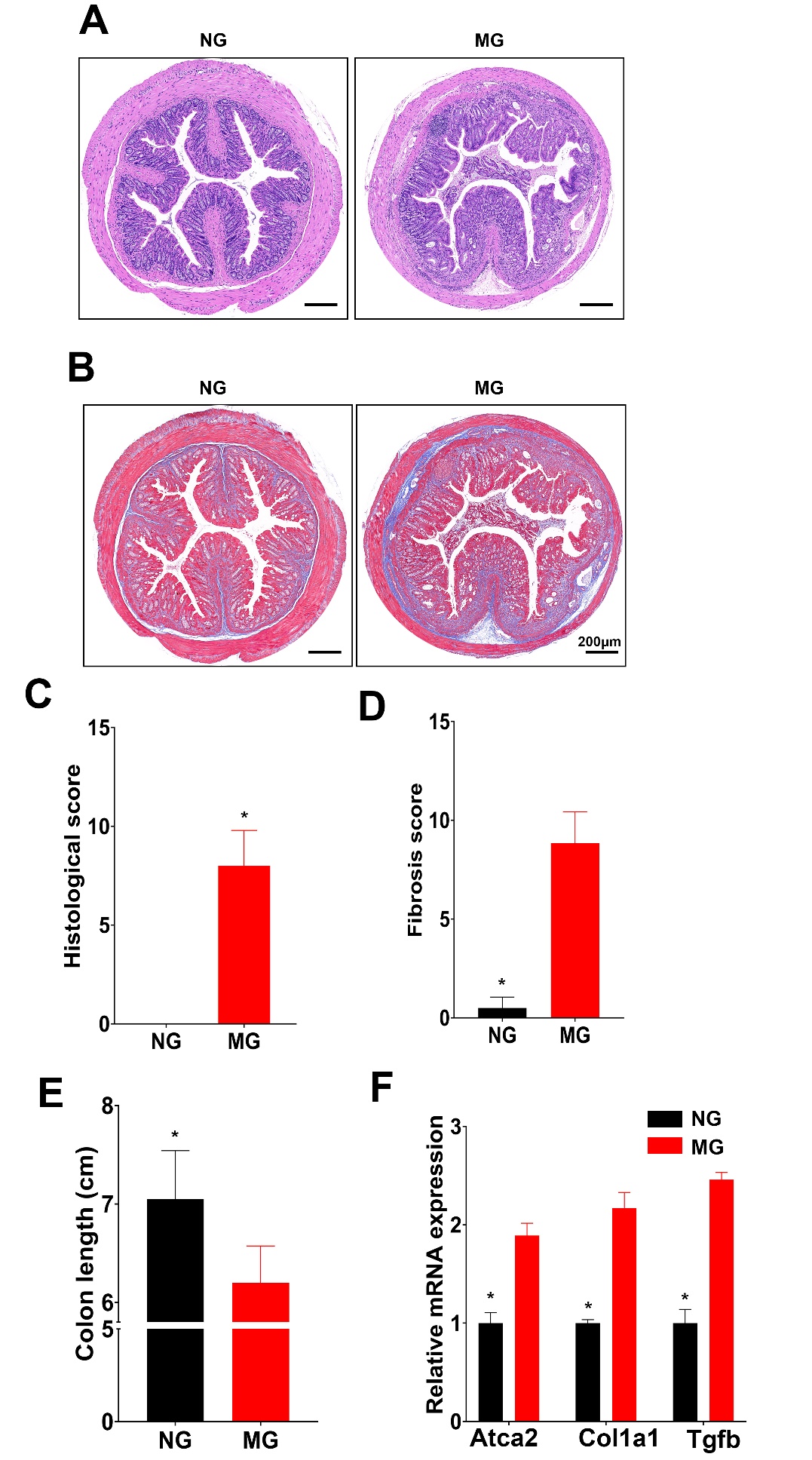
**

**Figure S2**

**
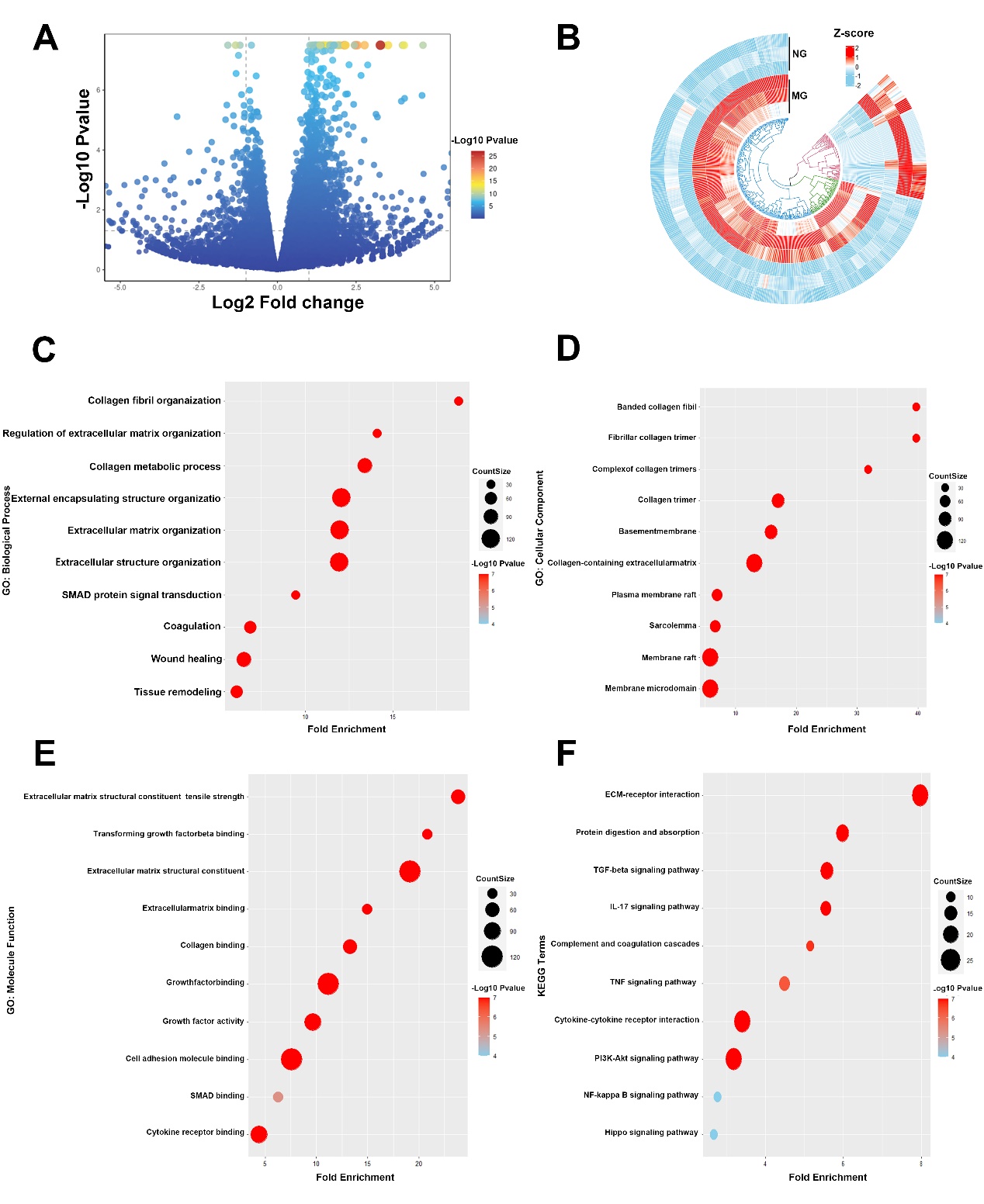
**

**Figure S3**

**
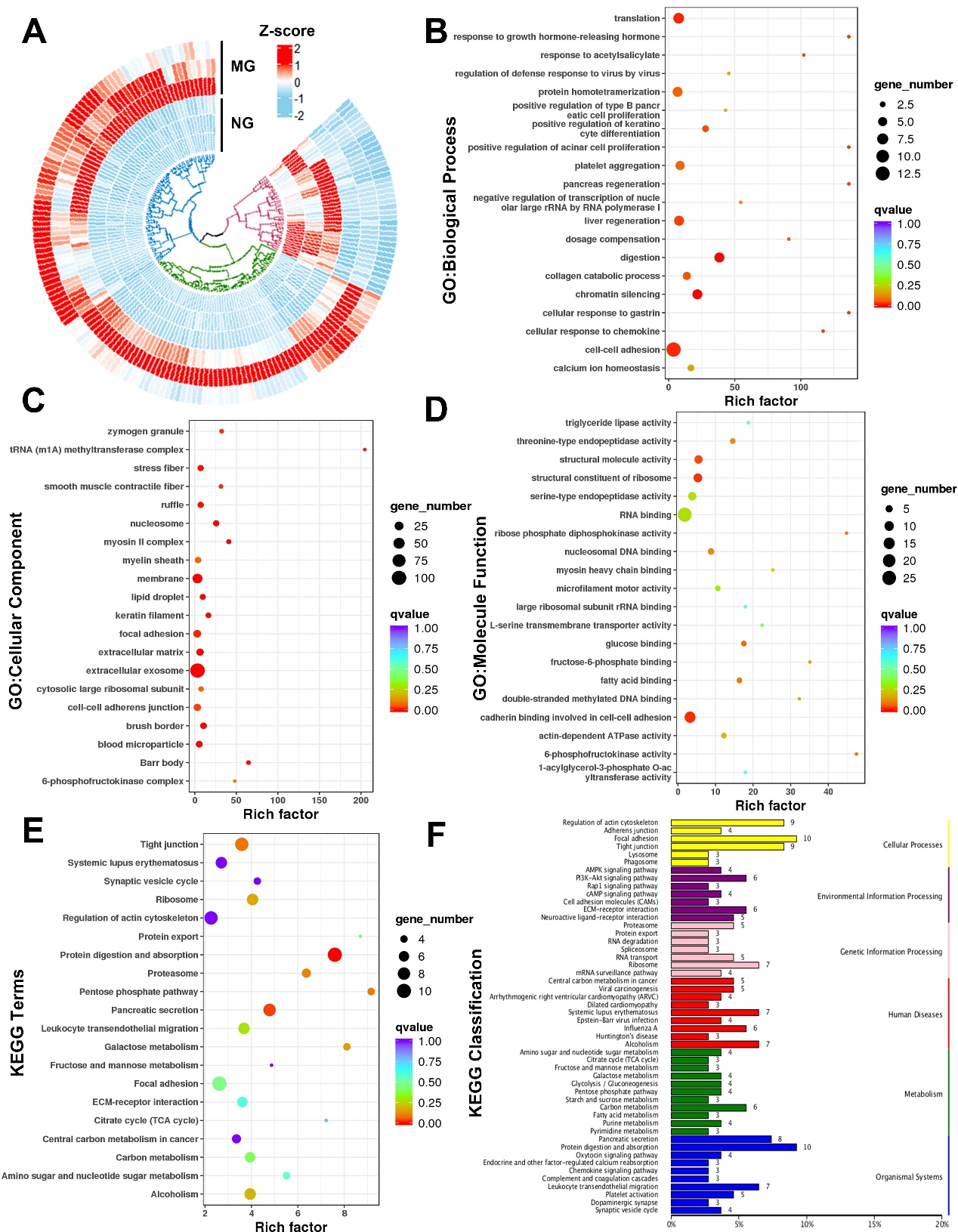
**

**Figure S4**

**
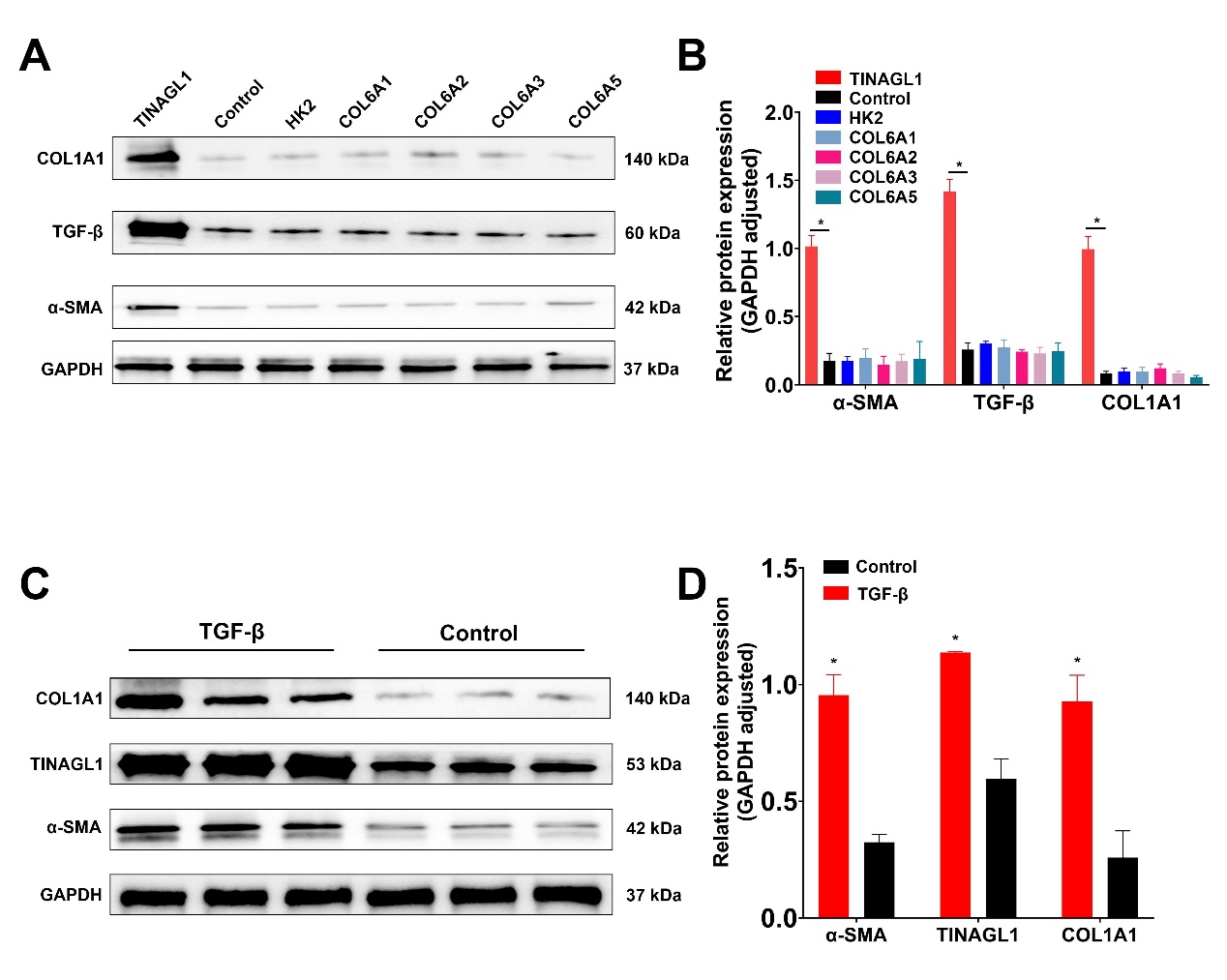
**

**Figure S5**

**
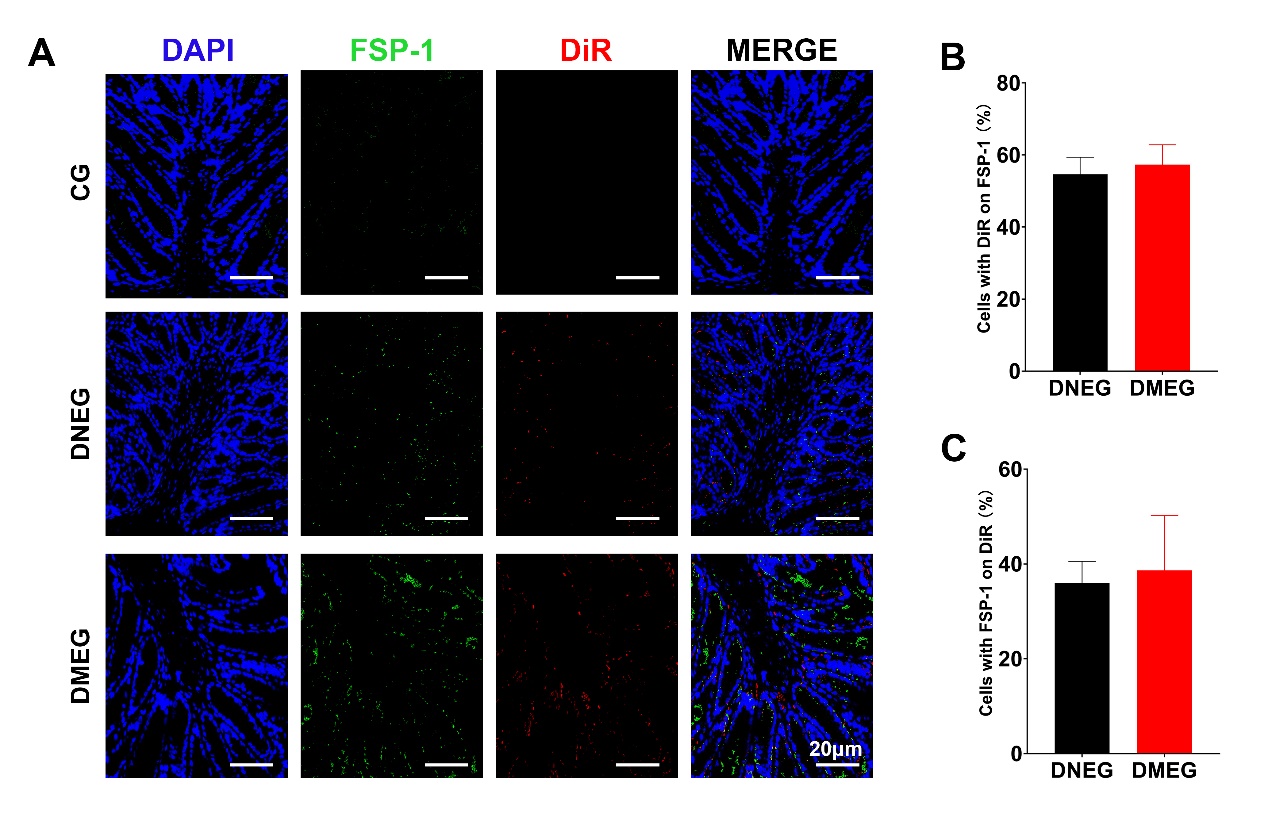
**

**Figure S6**

**
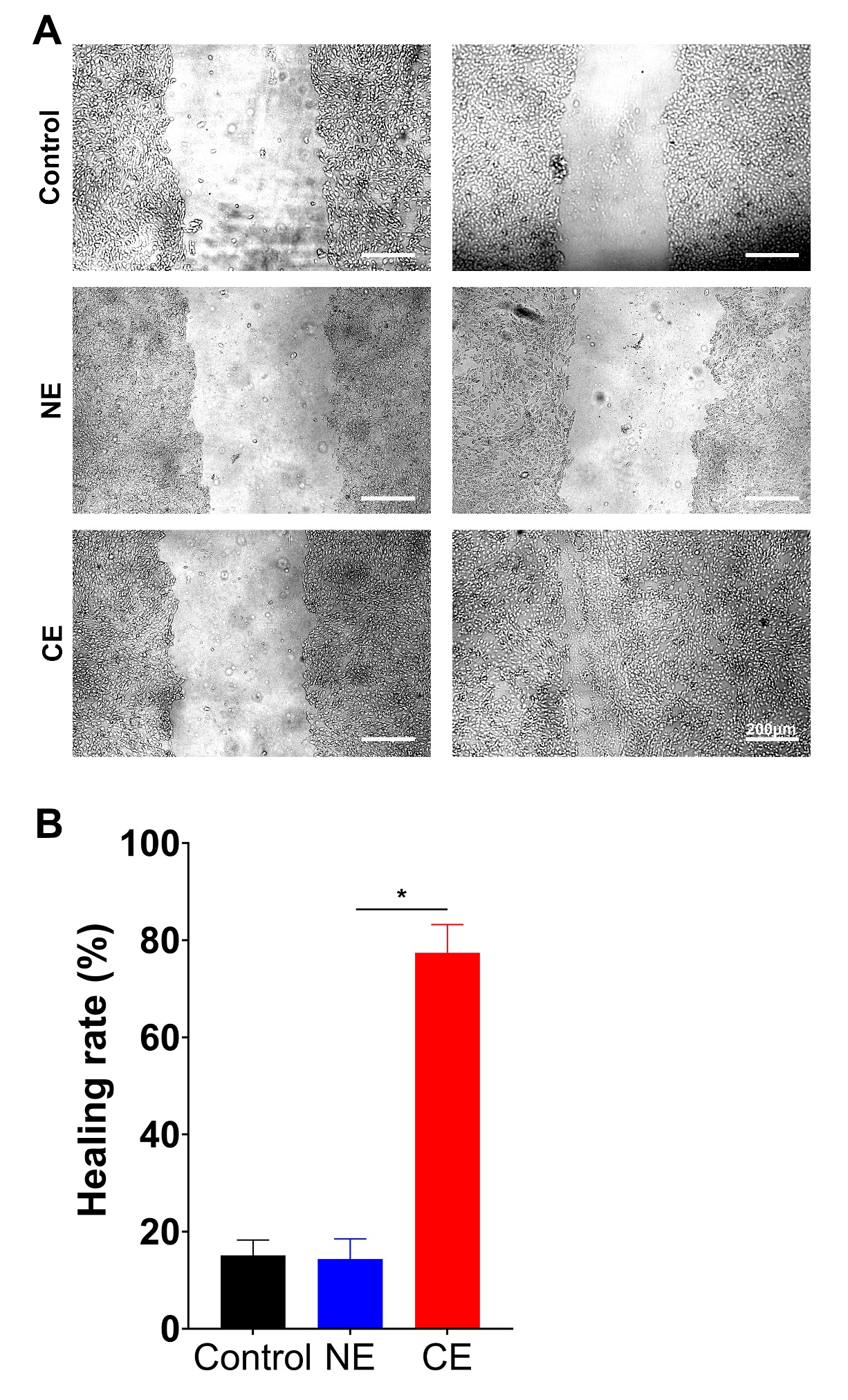
**

**Figure S7**

**
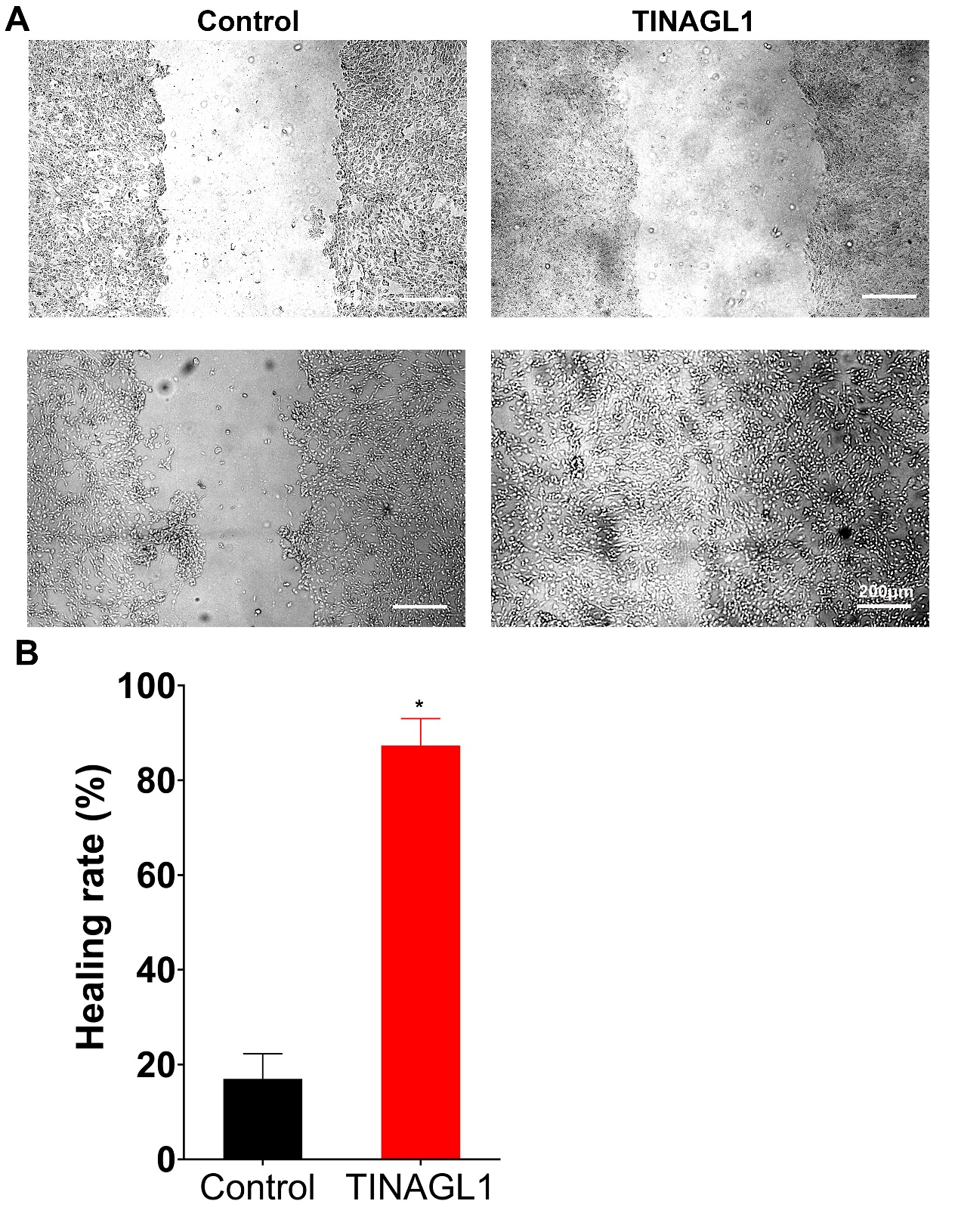
**

**Figure S8**

**
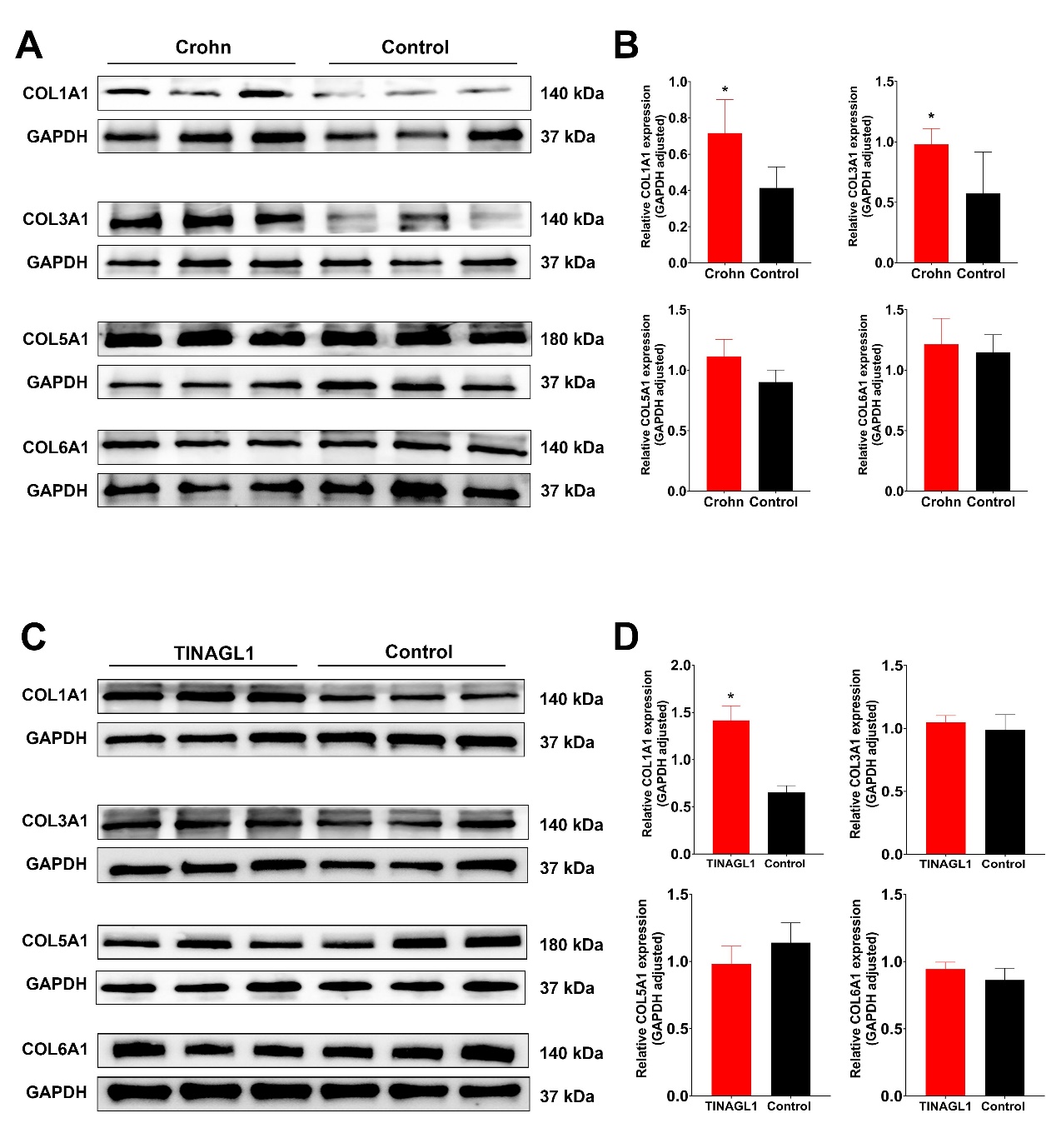
**

**Figure S9**


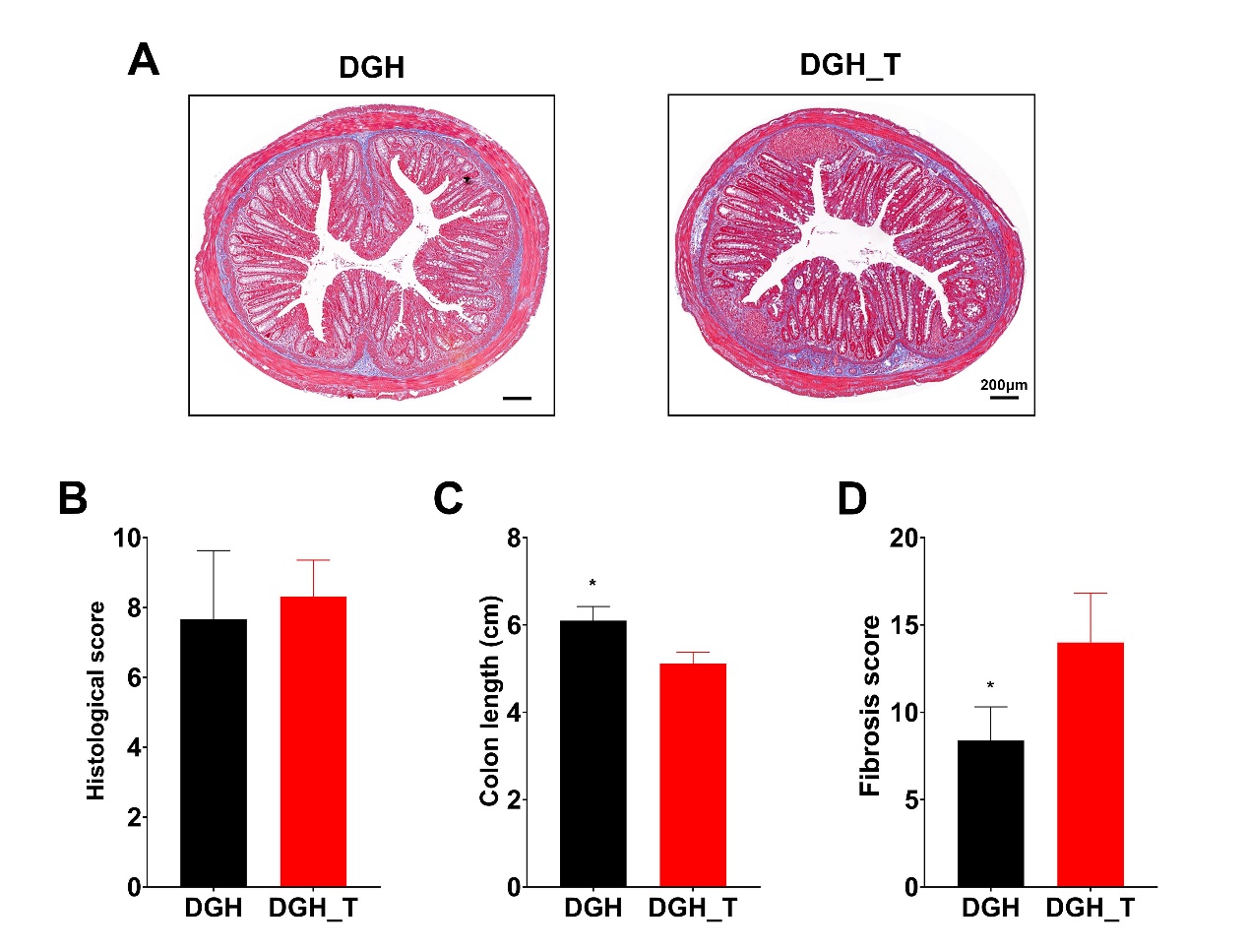


FIGURE LEGEND

**Figure S1. Histological and Molecular Characterization of DNBS-Induced Intestinal Fibrosis in Mice**

(A) Hematoxylin and Eosin (H&E) staining of colon tissues showing pronounced inflammation in the model group (MG) compared to the normal control group (NG), with images depicting histopathological changes (Scale bar: 200µm). (B) Masson's trichrome staining of colon sections illustrating significant fibrosis in MG with increased collagen deposition (Scale bar: 200µm). (C) Histological score analysis demonstrating higher scores in MG, reflecting severe inflammation and tissue damage. (D) Fibrosis score analysis showing increased fibrosis in MG. (E) Measurement of colon length, indicating shorter colons in MG, a consequence of fibrosis. (F) Gene expression analysis of fibrosis markers Acta2, Col1a1, and Tgfb, with higher expression in MG, consistent with fibrotic development, GAPDH used as an internal control. (**p* < 0.05, n=6, Statistical significance determined using a two-tailed Student's t-test).

**Figure S2. Transcriptomic Analysis of DNBS-Induced Intestinal Fibrosis in Mice** (A) Volcano plot depicting gene expression changes in the DNBS-induced mouse model, highlighting genes significantly upregulated or downregulated in the model group (MG) compared to normal controls (NG). (B) Circular heatmap showcasing the consistency of gene expression changes within the MG. (C-E) Gene Ontology (GO) enrichment analysis of differentially expressed genes, identifying significant enrichment in fibrosis-related Biological Processes (C), Cellular Components (D), and Molecular Functions (E). (F) Kyoto Encyclopedia of Genes and Genomes (KEGG) pathway analysis revealing the involvement of critical fibrosis-associated pathways in the MG. (n=3).

**Figure S3. Comprehensive Analysis of Differentially Expressed Proteins in Mouse Mesenteric Adipose Tissue-Derived Exosomes**

(A) Circular heatmap depicting the expression patterns of differentially expressed proteins between the MG and NG groups, showing consistent expression trends within each group. (B-D) Gene Ontology (GO) enrichment analysis of differentially expressed proteins, categorized into Biological Process (B), Cellular Component (C), and Molecular Function (D). These analyses highlight specific biological terms and pathways potentially involved in fibrogenesis. (E) KEGG pathway enrichment analysis of differentially expressed proteins, identifying key pathways implicated in the fibrotic process. (F) Classification of enriched KEGG pathways, providing a comprehensive overview of the molecular pathways potentially influenced by the differentially expressed proteins in MG exosomes. (n=3).

**Figure S4. TINAGL1 Mediates Fibrogenic Marker Expression in Human Primary Colonic Fibroblasts**

(A) Western blot analysis showing upregulation of fibrogenic markers (COL1A1, α-SMA, and TGF-β) in human primary colonic fibroblasts treated with recombinant TINAGL1, HK2, COL6A1, COL6A2, COL6A3, and COL6A5, compared to untreated control. (B) Quantitative analysis of the Western blots from (A); data normalized to GAPDH. (C) Western blot illustrating enhanced expression of TINAGL1 following treatment with recombinant human TGF-β.(D) Quantitative analysis of the Western blot data from (C), with results normalized to GAPDH. (n=6; Statistical significance determined by one way ANOVA, **p*<0.05).

**Figure S5. Assessment of Exosome Uptake by Colonic Fibroblasts in DNBS-Induced Mouse Models**

(A) Immunofluorescence images display the uptake of DiR-labeled exosomes (red) within colonic fibroblasts. FSP-1 staining (green) highlights fibroblast localization in the colon. The merged images show the successful absorption of DiR-labeled exosomes by fibroblasts in both DNBS with Model Group Exosomes (DMEG) and DNBS with Normal Group Exosomes (DNEG) groups (Scale bar: 20µm). (B and C) Quantitative analysis of the fluorescence intensity of DiR on FSP-1 and FSP-1 on DiR. This analysis reveals no significant difference in the uptake of labeled exosomes by fibroblasts between the DMEG and DNEG groups. (n=3, Statistical significance determined using a two-tailed Student's t-test).

**Figure S6. Wound Healing Assay in Primary Human Intestinal Fibroblasts Treated with Exosomes**

(A) Images from the wound healing assay showing the rate of wound closure in primary human intestinal fibroblasts treated with exosomes from the Control (NE), Crohn (CE) and control groups. (B) Quantitative analysis of the wound healing assay. The results indicate a significantly faster wound healing in cells treated with CE exosomes compared to those treated with NE exosomes. (n=6; Statistical significance determined by one way ANOVA, **p*<0.05).

**Figure S7. Wound Healing Assay in TINAGL1-Treated Primary Human Intestinal Fibroblasts**

(A) Photographic documentation of the wound healing assay, illustrating the closure of wounds in fibroblasts treated with recombinant TINAGL1 protein compared to the control group. Images captured at 24 hours post-treatment show the variation in wound closure rates between the two sets of fibroblasts. (B) Quantitative analysis of wound healing, revealing a significantly faster closure rate in fibroblasts treated with recombinant TINAGL1 protein. This enhancement in wound closure highlights the potent effect of TINAGL1 on fibroblast migration and wound healing capabilities. (n=6; Statistical significance determined using a two-tailed Student's t-test, **p*<0.05).

**Figure S8. Impact of TINAGL1 on Collagen Expression in Crohn's Disease Tissue and Treated Human Colonic Fibroblasts**

(A) Western blot analysis of collagen proteins (COL1A1, COL3A1, COL5A1, COL6A1) in narrowed colon sections from Crohn's Disease patients and matched control tissues. (B) Quantitative analysis of collagen protein expression levels from (A), normalized to GAPDH. (C) Western blot showing the effect of recombinant TINAGL1 protein on collagen expression in human primary colonic fibroblasts compared to control. (D) Quantitative analysis of collagen protein expression from (C), normalized to GAPDH. (n=6; Statistical significance determined using a two-tailed Student's t-test, **p*<0.05).

**Figure S9. Assessment of Fibrosis in Mice Treated with TINAGL1-Containing Hydrogel**

(A) Masson's trichrome staining of colon tissues from the DGH (control hydrogel) and DGH_T (TINAGL1-containing hydrogel) groups, illustrating differences in collagen deposition (Scale bar: 200µm). (B) Histogram of Histological score showing no significant difference in tissue damage between the DGH and DGH_T groups. (C) Bar graph of colon lengths indicating shorter colons in the DGH_T group, suggesting enhanced fibrosis. (D) Bar graph of Fibrosis scores highlighting increased fibrosis in the DGH_T group compared to the DGH group. (n=6; Statistical significance determined using a two-tailed Student's t-test, **p*<0.05).
